# Anthropometric Profile, Body Composition, and Game Performance of the Mexican Professional Basketball League Champion Team During the 2025–2026 Basketball Champions League Americas: A Cross-Sectional Study

**DOI:** 10.3390/jfmk11040386

**Published:** 2026-09-26

**Authors:** Carlos Abraham Herrera-Amante, Rodrigo Villaseca-Vicuña, Rodrigo Yáñez-Sepúlveda, Brenda Cecilia Jaime-González, José Raúl Hoyos-Flores, Exal Garcia-Carrillo, Matías Morán-Bravo, Boryi A. Becerra-Patiño, José Francisco López-Gil

**Affiliations:** 1 Universidad de Guadalajara, Centro Universitario de Tonalá, División de Ciencias de la Salud, Laboratorio de Evaluación y Cuidado del Estado Nutricio, Tonalá 45425, Jalisco, Mexico; carlos.amante@academicos.udg.mx; 2 Red Iberoamericana de Investigadores en Antropometría Aplicada, 04120 Almería, Spain; jose.hoyosfl@uanl.edu.mx; 3 Faculty of Education, School of Educational Sciences and Technology, Physical Education Pedagogy, Universidad Católica Silva Henríquez, Santiago 8280354, Chile; rvillaseca@ucsh.cl; 4 Faculty of Education and Humanities, School of Sport Sciences, Universidad Andres Bello, Viña del Mar 2520000, Chile; rodrigo.yanez.s@unab.cl; 5 Department of Health Research, Icen Cognis, 38370 La Matanza de Acentejo, Spain; 6 Sports Nutrition Department, Astros de Jalisco Basketball Club, Guadalajara 44210, Jalisco, Mexico; jaimebrendac@gmail.com; 7 Faculty of Sports Organization, Autonomous University of Nuevo Leon, San Nicolas de los Garza 66455, Nuevo Leon, Mexico; 8 Department of Physical Activity Sciences, Faculty of Education Sciences, Universidad Católica del Maule, Talca 3480112, Chile; exal.garcia@gmail.com; 9 Department of Physical Activity Sciences, Universidad de Los Lagos, Osorno 5290000, Chile; 10 Facultad de Medicina y Salud, Universidad Finis Terrae, Santiago 7501015, Chile; drmatiasmoran@gmail.com; 11 Facultad de Educación Física, Universidad Pedagógica Nacional, Bogota 480100, Colombia; babecerrap@pedagogica.edu.co; 12 Faculty of Sport Science, University of Murcia, 30100 Murcia, Spain; 13 School of Medicine, Universidad Espiritu Santo, Samborondon 092301, Ecuador; 14 Faculty of Health Sciences, Universidad Tecnológica Atlántico Mediterráneo—UTAMED, 29590 Málaga, Spain

**Keywords:** basketball, anthropometry, body composition, somatotype, bioelectrical impedance, phase angle, kinanthropometry, performance analysis, team sports

## Abstract

**Background and Objectives:** Basketball imposes a marked positional division of labour, and body size, somatotype and body composition are among the variables most consistently monitored in professional practice. Whole-roster studies pairing a complete kinanthropometric assessment with official competition statistics from the same competitive window are nevertheless scarce, and Latin American professional teams are almost absent from this literature. This study aimed (i) to describe the anthropometric profile, somatotype, body composition and field-test aerobic performance of the champion team of the Mexican professional league during the 2025–2026 Basketball Champions League Americas, and (ii) to explore, as a hypothesis-generating exercise, the association between these characteristics and per-game output from official box scores. We hypothesised a priori that any such association would be largely explained by playing position rather than by a direct effect of body build on performance. **Methods:** A cross-sectional census of the entire active roster (*n* = 18), assessed on a single day three days before the first of the eight games analysed, was carried out. Sixteen players completed a full International Society for the Advancement of Kinanthropometry (ISAK) profile with Heath–Carter somatotype and multi-frequency bioelectrical impedance analysis, 16 had official box-score data and 14 had both; 13 completed the 20 m multistage shuttle run. The shuttle-run result is reported as the maximal aerobic speed attained (km·h^−1^); the maximal oxygen uptake (VO_2_max) derived from it is a field-test estimate, used only as a within-team ranking and never as an absolute physiological value. Sixty Spearman correlations were pre-specified as exploratory and are reported with bootstrap and Fisher *z* 95% confidence intervals and Benjamini–Hochberg false-discovery-rate control. **Results:** Players were tall and lean (stature 194.6 ± 9.1 cm; body mass 92.8 ± 13.6 kg; body mass index (BMI) 24.4 ± 2.2 kg/m^2^; Σ8 skinfolds 82 ± 29 mm), with a balanced-mesomorph somatotype (2.5–4.5–3.0) and a high mean phase angle (8.8 ± 0.8°). Twelve correlations reached *p* < 0.05, but only one survived multiplicity control: maximal aerobic speed with rebounds per game (*ρ* = −0.86, *p* < 0.001, *q* = 0.018); this association disappeared when aerobic power was expressed in absolute terms (*ρ* = −0.17, *p* = 0.587), identifying body size as its source. Stature, body mass, Σ8 skinfolds and endomorphy were inversely associated with assists per game (*ρ* = −0.60 to −0.70; *p* = 0.006–0.022), and BMI, mesomorphy, ectomorphy and phase angle with field-goal percentage (*ρ* = −0.57 to +0.69; *p* = 0.007–0.039), but intervals were wide, *q* ≥ 0.078 for these associations, and field-goal percentage rested on an unknown number of attempts. **Conclusions:** This championship roster showed the tall, long-limbed, balanced-mesomorphic profile of the elite game and was markedly less endomorphic than Mexican athletic reference values. The a priori hypothesis was supported: every association was interpretable as an expression of playing position, and the single association surviving multiplicity control proved to be an artefact of body-size standardisation. Within-team anthropometry–output correlations should not be used to inform selection or playing-time decisions.

## 1. Introduction

Basketball is an intermittent, high-intensity team sport in which players repeatedly alternate short accelerations, decelerations, changes of direction, jumps and physical contacts with brief and incomplete recovery periods [1,2]. Systematic reviews of match-play have shown that both external and internal load are substantial and vary with competitive level [1,3], and that the most demanding passages of a game concentrate running intensities far above match averages into very short windows [4]. Critically, these demands are not distributed evenly across a roster: guards cover more distance and perform more high-intensity accelerations, whereas forwards and centres accumulate more jumps, contacts and low-speed actions in the confined space near the basket [5,6]. Positional specialisation is therefore embedded in the physical structure of the game itself.

Against this background, morphological and body-composition characteristics are among the most consistently monitored variables in applied basketball practice [7,8]. Stature, arm span and standing reach are stratified by playing position and discriminate selected from non-selected players in draft settings [9], and comparable stratification in body mass, stature and skinfold distribution has been reported in Spanish samples spanning professional leagues and junior national teams [10] and in Turkish professional squads [11], while physical-capacity profiles differ both by competitive division and by playing position [12]. At the highest international level, body height discriminates the better-placed national teams: players from top-16 International Basketball Federation (FIBA) World Cup teams were significantly taller at the point-guard, shooting-guard and small-forward positions [13]. Within a squad, centres and power forwards are consistently taller and heavier than guards, with correspondingly greater bone breadths and girths, whereas guards present the leanest and most linear builds [14]; the same positional architecture is already discernible in youth and high-school elite cohorts [15,16].

Standardised assessment is the prerequisite for any such comparison. The International Society for the Advancement of Kinanthropometry (ISAK) protocol provides an internationally accepted set of landmarks, instruments and procedures for surface anthropometry [17], and the Heath–Carter method converts these measures into endomorphy, mesomorphy and ectomorphy, which summarise relative adiposity, musculoskeletal robustness and linearity in a single comparable descriptor [18]. Somatotype has been used to characterise basketball players from youth to professional level [14,19,20,21] and, in the Mexican context, to compare athletes across a broad range of disciplines [22]. Surface anthropometry is increasingly combined with bioelectrical impedance analysis (BIA), which adds estimates of fat-free mass, skeletal-muscle mass and body water together with raw bioelectrical descriptors such as the phase angle [23,24,25]. The phase angle is interpreted as an index of cell membrane integrity and of the balance between extracellular and intracellular water [26,27,28]; it is higher in athletes than in non-athletes and, in some athletic populations, has been associated with strength and endurance performance [29,30]. No single technique is free from error in athletes, and pragmatic multi-method profiling remains the recommended approach in applied sport practice [24,31].

What remains far less clear is the extent to which these characteristics translate into measurable competitive output. Two largely separate literatures coexist: the first links anthropometry and fitness testing to selection outcomes, draft status, talent identification and competitive level, and consistently reports that height, wingspan and jump ability discriminate selected from non-selected players [9,32,33,34]; the second analyses game-related statistics to describe what distinguishes winning from losing teams, or one playing role from another, generally without reference to body composition [35,36,37,38,39]. Studies that connect a complete anthropometric and body-composition assessment to official box-score output at the level of individual players within a single high-level roster are uncommon, and the few available report modest and inconsistent associations [40]. Interpretation is further complicated by a structural feature of team rosters: within a homogeneous elite squad, between-player variation in body size is tightly coupled to playing role, so that any raw anthropometry–performance correlation is confounded by position by construction. Finally, most published profiles originate from European, North American or Asian competitions; Latin American professional teams and Mexican teams in particular remain under-represented, despite a growing body of applied kinanthropometric work in the region [41,42,43,44,45]. The gap addressed here is therefore threefold: whole-roster rather than positional-subgroup profiling; simultaneous acquisition of kinanthropometry, raw bioelectrical parameters and official competition output within a single competitive window; and a Latin American professional setting. The present work extends previous evidence by moving the analysis from group-level description to the individual player inside one elite roster, and by treating the structural entanglement between body size and playing role, rather than the correlation coefficients themselves, as the object of interpretation.

Therefore, this study had two aims: (i) to describe the anthropometric profile, somatotype, body composition and field-test aerobic performance of a professional basketball team assessed at the moment of its greatest competitive success, the champion of the Mexican professional league, competing internationally in the Basketball Champions League Americas, and (ii) to explore the association between these characteristics and per-game output derived from official competition statistics. Given the homogeneity and the small size of a single roster, the correlational component was pre-specified as exploratory and hypothesis-generating. The study tested one a priori hypothesis, stated here as a single testable proposition and resolved explicitly in Section 4.4 and Section 5: within a single elite professional roster, any association between anthropometric or body-composition variables and per-game competitive output is largely explained by (confounded with) playing position, which acts as a common cause of both body build and statistical role, rather than reflecting a direct effect of body size or composition on performance.

## 2. Materials and Methods

### 2.1. Study Design

This was a single-team, cross-sectional, observational study. The independent variables were anthropometric and body-composition descriptors; the dependent variables were per-game performance indicators derived from official competition statistics. All data collection took place on a single occasion, in one morning session on 25 January 2026, at the club’s training facility in Guadalajara, Mexico. This date fell three days before the team’s first game of the competition (28 January 2026), so that every player was assessed immediately before, and not during, the competitive window from which the performance data derive. That window comprised the eight games played by the club in the 2025–2026 Basketball Champions League Americas (BCLA). Recruitment was by whole-roster invitation: the club’s performance department invited every player registered on the first-team roster to attend the assessment session; no sampling, screening or selection of players was performed by the research team. The study is reported in accordance with the Strengthening the Reporting of Observational Studies in Epidemiology (STROBE) recommendations for cross-sectional research [46] (Table S1).

### 2.2. Setting and Ethical Approval

The study was conducted in Guadalajara, Mexico, during the 2025–2026 competitive season. It was carried out in accordance with the ethical principles for medical research involving human participants set out in the Declaration of Helsinki [47] and with the international guidelines for Good Clinical Practice. The Research Ethics Committee of the University of Guadalajara approved the protocol under code CEI-02-2024-07. All players provided written informed consent for the use and publication of their assessment and competition data. Because the club is identified by name and its competition roster is in the public domain, individual-level data cannot be regarded as fully anonymous; players and club management were informed of this limitation before consenting. No player name, jersey number, nationality or other direct identifier is reported, and data are presented without any label that links a data point to a named individual.

### 2.3. Participants

The roster comprised 18 male professional players from Astros de Jalisco. Inclusion criteria were (i) registration on the club’s first-team roster for the 2025–2026 BCLA at the time of assessment, (ii) clearance by the club’s medical department to train and compete without restriction, and (iii) written informed consent. Exclusion criteria were (i) inability to attend the assessment session, and (ii) removal from the competition roster before the analysed window. No player was excluded on clinical grounds.

No a priori sample-size calculation was performed, and none would have been informative. In elite team sport, the accessible population is not a sample but a census: the number of eligible athletes is fixed by the size of the active roster, and enrolling additional players, for instance from rival clubs during an international competition, is not feasible for either logistic or competitive reasons. We therefore assessed the entire available universe, that is, the full active roster, and we report the resulting precision transparently rather than a nominal power target. For orientation, with the 14 players who contributed paired anthropometric and box-score data, a two-tailed test at *α* = 0.05 reaches 80% power only for *ρ* ≥ 0.69 (*ρ* ≥ 0.73 for the 12 players with shuttle-run data). Effects smaller than this cannot be detected reliably in a study of this kind, which is precisely why the correlational component is framed as exploratory and why interval width, rather than statistical significance, is treated as the primary result.

The number of players contributing to each component differs, and the reasons are as follows (summarised in Figure S1). Of the 18 rostered players, 16 attended the assessment session and completed the full anthropometric and bioimpedance profile; the remaining two did not attend and were therefore never measured. Sixteen players accumulated official competition minutes and therefore have box-score data; two of the 16 assessed players recorded no competitive minutes in the analysed window. Fourteen players have both anthropometry and box-score data and constitute the analysis set for the correlational component. The 20 m multistage shuttle run was completed by 13 of the assessed players, 12 of whom also have box-score data; these 12 form the analysis set for every association involving aerobic performance. Mean age was 30.8 ± 4.5 years (range 23–40).

The 16 players who completed the anthropometric assessment were classified into three positional groups, guards (*n* = 9), wings/forwards (*n* = 1) and “bigs” (power forwards and centres; *n* = 6), on the basis of the position registered in the official competition roster. Point and shooting guards were pooled because both operate primarily on the perimeter with ball-handling and distribution responsibilities, and power forwards and centres were pooled as “bigs” because both operate mainly in and around the restricted area, where rebounding and short-range scoring are concentrated; this two-group structure follows the backcourt–frontcourt distinction used in positional analyses of match demands and box-score output [5,6]. Because the wing/forward group comprised a single player, the positional structure of this roster is effectively a guard–big dichotomy. Positional comparisons are therefore reported descriptively only, and no between-group statistical testing was undertaken; in addition, because a single athlete cannot constitute a group, the entire correlational analysis was repeated with that player excluded, as a pre-specified sensitivity analysis (Section 3.4 and Table S2).

### 2.4. Anthropometry and Body Composition

A full anthropometric profile was obtained following the ISAK protocol [17]. All measurements were taken by a single anthropometrist holding a current Level 2 accreditation from ISAK at the time of data collection, in accordance with the standards set out in the 2011 edition of the International Standards for Anthropometric Assessment [17]; because one accredited operator performed every measurement, inter-observer error does not apply to this dataset. Every site was measured in duplicate, with a third measurement and retention of the median when duplicates differed by more than 5% for skinfolds or 1% for girths, lengths and breadths. The intra-observer technical error of measurement, computed for this operator during the study period, was <3.4% for skinfolds and <0.91% for all other measurements. The variables measured were: two basic measures (body mass and stretch stature) together with sitting height and arm span; the eight skinfolds of the full ISAK profile (triceps, subscapular, biceps, iliac crest, supraspinale, abdominal, front thigh and medial calf); five girths (arm relaxed, arm flexed and tensed, waist, gluteal and calf); and four bone breadths (biacromial, biiliocristal, biepicondylar humerus and biepicondylar femur). The subset used to compute the Heath–Carter somatotype comprised stature, body mass, the triceps, subscapular, supraspinale and medial-calf skinfolds, the biepicondylar humerus and femur breadths, and the flexed-and-tensed arm and calf girths. All measurements were taken in the morning, in a fasted and euhydrated state, in minimal clothing, on the right-hand side of the body. Body mass was recorded with a digital scale accurate to 100 g (Seca^®^ 874, Hamburg, Germany) and stretch stature with a portable stadiometer accurate to 1 mm (Seca^®^ 213). Skinfolds were measured with a Slim-Guide^®^ skinfold calliper (Creative Health Products, Plymouth, MI, USA). Girths were measured with a non-extensible metallic tape 0.7 mm thick, and lengths and breadths with professional anthropometric equipment accurate to 1 mm (SmartMet Kinanthropometric Assessment^®^, Guadalajara, Jalisco, Mexico).

Somatotype was computed with the Heath–Carter anthropometric method (endomorphy, mesomorphy and ectomorphy) and represented on a somatochart [18,23]. Rather than reproduce the full set of equations here, we note that all somatotype components and the derived anthropometric indices were calculated with the specialised software ISAK Metry version 2.1.4 (International Society for the Advancement of Kinanthropometry [ISAK], Murcia, Spain), which implements the standard Heath–Carter algorithms exactly as published by Carter and Heath [18] from the input measurements listed above; no equation was modified, and no locally derived coefficient was applied. Additional body-composition descriptors included the sum of the eight ISAK skinfolds (Σ8), body mass index (BMI), and fat-free-mass index (FFMI = fat-free mass/stature^2^), which expresses fat-free mass independently of stature and facilitates comparisons with athletic reference values [48,49]. Because bioimpedance-derived body-composition estimates are dependent on the measurement technique and data-processing procedures [50], the bioimpedance export underwent a formal data-quality audit (Table S3). This audit was prompted by the observation that the implied fat mass (FM) had been returned as zero or a negative value (≤ 0.00 kg) for five of the sixteen assessed players, yielding physiologically impossible values. We therefore applied a systematic range- and coherence-check to every bioimpedance-derived field, rather than to FMI alone. Three checks were run: (i) a physiological range check on each variable; (ii) a mass-balance check, in which fat mass implied by the export (body mass minus fat-free mass) was required to be positive; and (iii) an internal-consistency check, in which BMI was required to equal FMI + fat-free-mass index (FFMI). The audit showed that the problem was not confined to FMI. Implied fat mass was zero or negative in 5 of the 16 players (i.e., fat-free mass equalled or exceeded total body mass) and below 8% of body mass in 9 of the 16, and BMI differed from FMI + FFMI by more than 0.1 kg/m^2^ in 8 of the 12 players for whom a non-zero FMI had been recorded. The fat/fat-free partition produced by this export is therefore not trustworthy in this sample. Accordingly, fat mass, percentage body fat, FMI and FFMI were all treated as unusable and were removed from the manuscript: they are neither reported descriptively nor entered into any analysis. Raw bioelectrical parameters were affected differently. The phase angle is computed directly from measured resistance and reactance and does not depend on the fat/fat-free model, and it was recorded within the plausible physiological range in every player, including the players with missing or zero FMI in the export (Table S3); it was therefore retained, although with the interpretative caution set out in Section 4.2. Adiposity is characterised throughout this manuscript by Σ8 skinfolds and endomorphy, which are measured directly on the participant and do not depend on any bioimpedance model.

Multi-frequency bioelectrical impedance analysis was performed with a seca mBCA 525 8-electrode device (Seca^®^, Hamburg, Germany) operating at eight frequencies (1–500 kHz); data were exported with seca analytics 115 software. Standardisation comprised a 6–8 h overnight fast, bladder voiding immediately before measurement, abstention from exercise for 12 h and from strenuous exercise for 24 h, abstention from alcohol and caffeine, and 10 min of quiet supine rest before the measurement. For the reasons set out above, the only BIA-derived variable retained in this manuscript is the phase angle, which is a raw bioelectrical parameter independent of predictive equations and reflects the extracellular-to-intracellular water balance and body cell mass directly [27,28,51,52]. Estimates of fat mass, fat-free mass, skeletal muscle mass and every index derived from them were discarded.

Aerobic performance was assessed with the 20 m multistage shuttle run [53,54], performed on an indoor hardwood court immediately after a standardised 10 min warm-up. Players ran between two lines 20 m apart, pacing themselves to an audio signal; the initial speed was 8.5 km·h^−1^ and increased by 0.5 km·h^−1^ at each subsequent one-minute stage. The test was terminated when a player failed to reach the line in time on two consecutive occasions or stopped voluntarily; strong verbal encouragement was given throughout, and players walked freely for 5 min afterwards before a passive cool-down. Testing was performed at the end of the assessment session, after all anthropometric and bioimpedance measurements, so that the latter were unaffected by exercise.

The primary outcome of the test reported here is the directly observed one: the maximal aerobic speed attained, that is, the running speed corresponding to the last stage completed (km·h^−1^). Maximal oxygen uptake was additionally estimated from that speed with the adult equation of Léger et al. [54], VO_2_max = 6.0 × speed − 24.4. We report the estimate for comparability with the literature, but we do not analyse it as a physiological quantity, for two reasons. First, the values obtained exceed laboratory-measured values reported for basketball players of comparable or higher competitive level (Section 4.3), and the equation is applied well outside the anthropometric range in which it was derived; the estimate is therefore not valid in absolute terms for athletes of this stature and mass. Second, because the estimate is a strictly increasing linear function of the measured speed, every rank-based statistic computed on it is numerically identical to the statistic computed on the speed itself. All analyses in this manuscript are therefore reported on the maximal aerobic speed, which is a measured quantity rather than a model output; the corresponding VO_2_max estimate appears in Table 1 for descriptive comparison only, is interpreted exclusively as a within-squad ordinal ranking, and is never compared quantitatively with gas-analysis values. Because relative aerobic expression is itself a function of body size, a size-corrected sensitivity analysis was pre-specified and is reported in Section 3.4.

### 2.5. Game Statistics

Performance data were the official box scores of the 2025–2026 Basketball Champions League Americas (BCLA), published by FIBA on the competition’s official statistics portal (https://www.fiba.basketball/es/events/basketball-champions-league-americas-25-26/teams/astros-de-jalisco#statistics; accessed on 20 February 2026). The competition window analysed comprises the club’s full participation in that edition, a six-game group phase followed by a best-of-three quarter-final series, eight games in total, so that per-game values derive from a small number of team games and, for players with limited rotation, from fewer still (median 7.5 games per player, range 1–8).

Season totals for each player (games played, minutes, points, rebounds and assists) were extracted independently by two members of the research team and cross-checked against one another; discrepancies were resolved by returning to the official portal. This verification step was prompted by a formatting fault in the source spreadsheet: several per-game (mean) fields had been silently converted by the spreadsheet application into date values, which was detected when routine range checks returned per-game figures outside any plausible basketball range and inconsistent with the corresponding season totals. Because the season totals themselves were intact and matched the official portal, all per-game values used here were recomputed directly from those verified totals divided by games played, and the corrupted mean fields were discarded. The field-goal percentage was taken from the intact percentage field and was likewise cross-checked against the portal.

The performance indicators analysed are defined as follows. Points, rebounds (total, i.e., offensive plus defensive), assists and minutes per game are the corresponding season total divided by games played. Field-goal percentage is the proportion of field-goal attempts made, expressed as a percentage, as published in the official box score. A transparent composite variable, the combined points, rebounds and assists per game, was defined as the arithmetic sum of those three per-game values. It is used as a summary of aggregate offensive and rebounding volume and is not a validated efficiency index; because steals, blocks and turnovers were unavailable, and because the sum is unweighted, it conflates playing opportunity with productivity, and we therefore avoid the term “game-impact index” [35,39]. One caveat applies from the outset to field-goal percentage: the number of shot attempts underlying each player’s percentage could not be recovered from the extracted fields, so no minimum-attempt eligibility criterion could be applied, and percentages based on few attempts are inherently unstable. Associations involving this variable are consequently the least reliable in the analysis and are flagged as such wherever they appear.

### 2.6. Statistical Analysis

Analyses were performed in Python 3.12 (pandas, NumPy and SciPy) with a fixed random seed (42) for reproducibility. Continuous variables are summarised as mean ± standard deviation (SD) together with the median and interquartile range (IQR), which are reported alongside one another because several per-game distributions are markedly skewed, and with the range and the number of players contributing data. Given the small sample and the non-normality of several distributions, associations between anthropometric and performance variables were quantified with Spearman’s rank correlation coefficient (*ρ*); 95% confidence intervals were obtained by bias-corrected and accelerated (BCa) bootstrap resampling (2000 iterations). Because BCa intervals become unstable when the acceleration constant is estimated from 12–14 observations, and may then extend to the boundary of the parameter space, a second interval was computed for every reported coefficient by the Fisher *z* transformation with the Bonett–Wright standard error for Spearman’s *ρ*, SE = √[(1 + *ρ*^2^/2)/(*n* − 3)]. The two intervals are reported side by side, and agreement between them is treated as the criterion for regarding an estimate as reasonably stable. All analyses were complete-case; no imputation was performed.

Because the number of players is small and the number of candidate associations is large, the correlational analysis is explicitly exploratory. Ten anthropometric and body-composition variables were crossed with six per-game output variables, giving 60 correlation tests. Multiplicity was addressed in two ways. First, the false discovery rate across the full set of 60 tests was controlled with the Benjamini–Hochberg procedure [55], and the resulting *q* value is reported for every coefficient discussed; a coefficient is described as surviving multiplicity control only when *q* < 0.05. Second, and more importantly, effect estimates and their uncertainty, rather than the *p* < 0.05 threshold, are treated as the primary result throughout. We deliberately do not compare the observed number of nominally significant results with the number expected by chance: the 60 tests are strongly dependent, because the anthropometric variables are correlated with one another and the performance outcomes likewise, so the binomial expectation under 60 independent null tests is not a meaningful benchmark and could be misread as indirect evidence that the overall pattern is real. No magnitude-based inference or qualitative probabilistic descriptor was used, as such procedures inflate false-positive rates and are not recommended [56,57]; interval width is treated as the primary indicator of estimate precision [58].

Two sensitivity analyses were pre-specified. (i) Because the wing/forward group comprised a single player, all 60 correlations were recomputed with that player excluded (Table S2). (ii) Because relative aerobic expression (per kilogram of body mass, or equivalently running speed in a weight-bearing test) is systematically higher in smaller athletes, every association involving aerobic performance was recomputed with aerobic power expressed in absolute terms (L·min^−1^, obtained as the estimated relative value multiplied by body mass), and additionally with a rank-based partial correlation controlling for body mass (Table S4). This size correction for the aerobic variable is reported in Section 3.4. The complete 10 × 6 coefficient matrix is displayed in full in Table S5; Table 2 lists the coefficients reaching nominal *p* < 0.05, together with their *q* values. No position-adjusted or position-stratified analysis was performed: with nine guards, six bigs and a single wing/forward, partial or stratified estimates would be based on subgroups too small to support inference, and the resulting coefficients would be at least as unstable as the unadjusted ones. The role of playing position is therefore discussed as an interpretation of the observed pattern and not as a tested effect.

## 3. Results

### 3.1. Anthropometric and Body-Composition Profile

Descriptive characteristics are presented in Table 1. The roster was tall, long-limbed and lean, with a low-to-moderate skinfold sum and a mean BMI within the normal range. Arm span exceeded stature by 4.8 cm on average. Because several per-game distributions are skewed, with assists ranging from 0.0 to 4.6 and rebounds from 0.5 to 14.0, medians and interquartile ranges are reported alongside means and SDs. For every per-game variable, the median lies below the mean (points 6.3 [4.1–14.3] versus 8.8 ± 5.6; rebounds 2.9 [2.2–5.6] versus 4.1 ± 3.3), reflecting the right skew produced by a small number of high-usage players in a roster where playing time was very unevenly distributed (median 7.5 games played per player, range 1–8). Aerobic performance is reported as the maximal aerobic speed attained in the shuttle-run test (15.3 ± 1.2 km·h^−1^; median 15.2 [14.3–16.6]; range 13.7–17.0); the corresponding VO_2_max estimate (67.4 ± 7.1 mL·kg^−1^·min^−1^) is shown for descriptive comparison only and is discussed critically in Section 4.3. The mean phase angle was 8.8 ± 0.8°, a high value whose interpretation is qualified in Section 4.2; fat-free-mass index, fat-mass index and percentage body fat are not reported, for the reasons given in Section 2.4.

### 3.2. Somatotype

The mean somatotype was balanced mesomorph (2.5–4.5–3.0), with mesomorphy the dominant component, indicating a robust musculoskeletal build with moderate adiposity and linearity (Figure 1). Individual somatotypes were dispersed across the chart, reflecting the heterogeneity of body builds required by different playing roles within the same squad.

### 3.3. Profile by Playing Position

Anthropometric and body-composition descriptors differed markedly across positional groups (Figure 2, Table S6). Bigs were taller (203.0 ± 5.7 versus 188.7 ± 6.3 cm) and heavier (107.6 ± 9.1 versus 82.7 ± 4.2 kg) than guards and had a higher mean skinfold sum (92.1 ± 19.0 versus 78.0 ± 34.3 mm); the Σ8 distributions nevertheless overlapped substantially, and the two highest individual values in the roster belonged to guards, so leanness was not a uniform property of the backcourt. Mean phase angle was slightly lower in bigs than in guards (8.4 ± 0.9° versus 9.0 ± 0.8°), with fully overlapping ranges. Maximal aerobic speed was higher in guards (15.7 ± 1.2 km·h^−1^, *n* = 7) than in bigs (14.6 ± 1.1 km·h^−1^, *n* = 5), the direction expected when a weight-bearing running test is performed by athletes differing by approximately 25 kg in body mass. Because the number of players within each positional group was very small, and the wing/forward group comprised a single player (guards *n* = 9; wings/forwards *n* = 1; bigs *n* = 6), these comparisons are presented descriptively and were not tested statistically.

### 3.4. Associations Between Anthropometry and Game Output

Among the 14 players with both a complete anthropometric assessment and box-score data (12 for analyses involving aerobic performance), 12 of the 60 correlations tested reached nominal *p* < 0.05 (Figure 3 and Table 2), but only one survived Benjamini–Hochberg control of the false discovery rate: maximal aerobic speed with rebounds per game (*ρ* = −0.86, *p* < 0.001, *q* = 0.018). Four further coefficients lay between *q* = 0.05 and *q* = 0.10—stature, Σ8 skinfolds and endomorphy with assists per game, and BMI with field-goal percentage (*q* = 0.078 in each case)—and every remaining coefficient had *q* ≥ 0.11. In substantive terms, taller and heavier players and those with higher skinfold sums and endomorphy recorded fewer assists per game; higher BMI and mesomorphy, and lower ectomorphy and phase angle, accompanied a higher field-goal percentage; and a higher maximal aerobic speed accompanied fewer rebounds, fewer points and fewer minutes per game. Four of the twelve associations involve field-goal percentage, which, as stated in Section 2.5, was computed over an unknown and, for low-minute players, certainly small number of shot attempts; these four are the least reliable estimates reported here and are presented as exploratory observations rather than as findings. These directions are consistent with a single underlying pattern in which body size and composition track playing position, which in turn determines the statistical role a player fills (Figure 4); this reading is developed in Section 4.4 and was not tested formally.

Precision was low throughout. Several BCa intervals extended to the boundary of the coefficient (stature × assists per game: *ρ* = −0.70, 95% CI −1.00 to +0.01) or reached zero at one limit (mesomorphy × field-goal percentage: *ρ* = +0.56, 95% CI −0.03 to +0.87), behaviour that reflects the instability of the acceleration estimate at this sample size rather than genuine information about the parameter. The Fisher *z* intervals, which are not subject to that instability, were narrower at the extremes but included zero for two of the twelve coefficients (maximal aerobic speed × minutes per game and mesomorphy × field-goal percentage), and their lower bounds lay within 0.06 of zero for three further coefficients (body mass × assists per game, phase angle × field-goal percentage and ectomorphy × field-goal percentage). Read together with the false-discovery-rate results, this means that eleven of the twelve nominally significant coefficients are compatible with underlying effects ranging from negligible to very large, and that none of them should be treated as an established finding.

Both pre-specified sensitivity analyses were run. Excluding the single wing/forward left the pattern unchanged: the ranking of coefficients was preserved, the same single association survived false-discovery-rate control (*ρ* = −0.89, *p* < 0.001, *q* = 0.012), no coefficient changed direction, and the assists- and BMI-related coefficients became marginally stronger (|ρ| = 0.60–0.74) rather than weaker; no reported association therefore depends on retaining one unclassifiable player (Table S2). The size correction of the aerobic variable was far more consequential. Running speed in a weight-bearing shuttle test, and the relative VO_2_max derived from it, are both inversely related to body mass by construction [59]. When aerobic power was instead expressed in absolute terms (L·min^−1^), its association with rebounds per game disappeared entirely (*ρ* = −0.17, *p* = 0.587; Figure 4b), whereas its association with assists per game strengthened (*ρ* = −0.76, *p* = 0.004), the mirror image of the relative expression. A rank-based partial correlation adjusting for body mass retained a negative coefficient for rebounds (partial *ρ* = −0.84, *p* = 0.001), which is expected, since ranking players by running speed within a roster reproduces much of the backcourt–frontcourt split that body mass alone does not capture. The one association that survived multiplicity control is therefore an expression of body size and of the way aerobic capacity is standardised to it, and not evidence that aerobic fitness is detrimental to rebounding (Table S4).

## 4. Discussion

This study profiled a professional basketball team during its participation in a continental club competition and related a complete anthropometric assessment to official game output. The roster presented the tall, long-limbed, lean and predominantly mesomorphic profile expected of high-level basketball, together with a phase angle at the very top of the range described for athletes and a field-test aerobic result that, as discussed below, exceeds what shuttle-run prediction can plausibly support. Three findings organise the discussion that follows. First, the morphological profile is the substantive, reproducible contribution of this work, and it is markedly less endomorphic than the Mexican athletic reference. Second, of 60 exploratory correlations, only one survived multiplicity control, and a pre-specified size correction showed that this single survivor was an artefact of expressing aerobic capacity per unit of body mass. Third, and consequently, the a priori hypothesis stated at the end of Section 1 is supported: within this roster, every anthropometry–output association is interpretable as an expression of playing position, and none can be attributed to a direct effect of body size or composition on performance. Section 4.4 returns to that verdict explicitly.

### 4.1. Anthropometric and Somatotype Profile in Context

The mean stature (194.6 ± 9.1 cm) and body mass (92.8 ± 13.6 kg) of this roster fall between the positional means reported in a Spanish sample of 110 players drawn from professional leagues and junior national teams (guards 182.3 cm and 79.6 kg; forwards 195.7 cm and 91.0 kg; centres 204.1 cm and 104.6 kg) [10], which is precisely what a whole-roster mean should look like when the three positional groups are pooled. Comparable anthropometric stratification by position has been described in Turkish professional [11] and Polish professional [14] squads, in National Basketball Association (NBA) draft combine cohorts [9] and at an earlier developmental stage in Korean elite high-school athletes [16]; a parallel stratification in physical capacity, rather than in morphology, has been documented across competitive divisions and playing positions [12]. Body height also discriminates the better-placed national teams at the FIBA World Cup, where players from top-16 teams were significantly taller at the point-guard, shooting-guard and small-forward positions [13]. The mean arm span exceeding stature by approximately 5 cm is likewise consistent with the selection advantage attributed to relative limb length and standing reach in this sport [9,14,15].

The mean somatotype (2.5–4.5–3.0, balanced mesomorph) is almost identical to the 2.26–4.57–3.04 reported by Gryko et al. [14] for adult professional male players, reinforcing the notion of a stable morphological signature at the professional level. It is, however, notably less endomorphic than the profile most frequently observed in Mexican male athletes across 43 disciplines, in which the endomorphic mesomorph category predominates (52.4%) and only 17.6% are classified as balanced mesomorphs; that same national study concluded that Mexican athletes present a higher endomorphic component than their international counterparts [22]. Read against this national reference, the comparatively low endomorphy of the present championship roster (2.5 ± 1.0) is one of its more distinctive features and aligns with elite profiles described elsewhere [19,20,21]. Whether this reflects selection, training history, nutritional support or the international level of competition cannot be determined from a cross-sectional single-team design.

### 4.2. Bioelectrical Impedance and Phase Angle

The mean phase angle of this roster (8.8 ± 0.8°; range 6.9–10.1°) exceeds the 95th percentile of reference values derived from 1658 male athletes, in whom the median for team-sport athletes lies close to 7° [60], and approaches the highest group means in the literature, reported in bodybuilders on the day of competition. Phase angle is consistently higher in athletes than in non-athletes and reflects body cell mass and the balance between extracellular and intracellular water [26,27,28,61]. We nevertheless now regard an instrument- or protocol-related contribution as the more probable explanation, and we say so before offering any physiological reading. The decisive consideration is internal: the same export, from the same device and session, produced a fat/fat-free partition that failed three independent coherence checks (Section 2.4), including an implied fat mass of zero or less in 5 of 16 players. An export that is demonstrably mis-calibrated in one output cannot be assumed to be calibrated in another, even when the second output is a raw parameter, because resistance and reactance enter both. A value nearly a full degree above the 95th percentile of a 1658-athlete reference is, on its own, more readily explained by device, frequency, electrode-configuration or posture differences than by a genuine physiological peculiarity of a 16-player squad. We therefore report the phase angle as a device-specific descriptive observation; we do not present it as a distinctive physiological feature of this team, and we advise against comparing it with values obtained using other analysers. Its relationship with performance is in any case unsettled: a systematic review of 35 studies found no clear relationship between phase angle and sport performance [26], although later work in adolescent female basketball players reported associations of whole-body and upper-limb phase angle with sprint and endurance capacity after adjustment for age and lean soft tissue [29], and a cross-sport meta-analysis reported associations with limb strength [30].

In the present data, the only nominally significant association involving phase angle was a negative correlation with field-goal percentage (*ρ* = −0.57; BCa 95% CI −0.83 to −0.13; Fisher *z* 95% CI −0.86 to −0.02; *q* = 0.202), a direction for which no physiological rationale exists and which we attribute to positional confounding, to the instability of a percentage computed over few attempts, and to sampling variability. A direct comparison with the only closely comparable study reinforces this reading. Bertuccioli et al. [62] studied a professional basketball squad of almost identical size (*n* = 14) and reported that BMI was directly proportional to the number of assists, whereas in the present roster stature, body mass, Σ8 skinfolds and endomorphy were all inversely associated with assists per game, and BMI showed no association with that outcome. Three explanations can be weighed. League level is unlikely to be decisive: both squads are professional, and there is no mechanism by which competitive standard would reverse the sign of a size–assists relation. Roster composition is a stronger candidate: the assists–size relation within a team is generated by how many ball-dominant guards a squad carries and how minutes are distributed among them, and two rosters differing in the guard-to-big ratio, or in whether a large point-forward handles the ball, will generate opposite gradients from identical physiology. Low power, however, is the most parsimonious explanation and is sufficient on its own: with 13–14 players, the 95% interval around a coefficient of this magnitude spans most of the parameter space (Table 2), so two independent studies of this size can readily produce coefficients of opposite sign by sampling variation alone, without any substantive difference between the populations. The contrast is therefore best read not as a discrepancy requiring a physiological explanation but as an illustration of what single-team correlational designs can and cannot resolve.

### 4.3. Field-Test Aerobic Performance

Players attained a maximal aerobic speed of 15.3 ± 1.2 km·h^−1^ (range 13.7–17.0), which under the adult equation of Léger et al. [54] corresponds to an estimated VO_2_max of 67.4 ± 7.1 mL·kg^−1^·min^−1^ (maximum 77.6). Those estimated values are implausibly high for this population. Directly measured VO_2_max in Italian regional-level basketball players was 50.3 ± 4.0 mL·kg^−1^·min^−1^ in senior and 60.9 ± 6.3 mL·kg^−1^·min^−1^ in junior players [63], and values above 70 mL·kg^−1^·min^−1^ are characteristic of endurance specialists rather than of athletes of 195 cm and 93 kg. A higher competitive level cannot reasonably account for a difference of this size. Two sources of error are plausible and are not mutually exclusive. The first is prediction error: the shuttle-run equations were not derived in athletes of this stature and mass, prediction accuracy in Mexican samples improves substantially when adiposity indicators are added to the equation [64], and reliability varies with population and testing conditions [65], even though the test correlates strongly with directly determined VO_2_max [66]. The second is the measured speed itself: a maximal aerobic speed of 17.0 km·h^−1^ in a 108 kg athlete is at the very top of what has been reported for team-sport players, and administration factors, court surface and turning technique, audio-signal calibration, or pacing in a small group with strong verbal encouragement, can inflate the last stage completed independently of any equation. Reporting the raw speed rather than only the derived estimate makes this second possibility visible and auditable, which is the main reason we now do so. For the purposes of this study, the consequence is the same in either case: the aerobic result is used only as a within-squad ordinal ranking, its absolute magnitude should not be quoted as a physiological characteristic of the roster, and comparison with laboratory-derived values is not warranted.

The associations between maximal aerobic speed and rebounds (*ρ* = −0.86), points (−0.69), the combined index (−0.69) and minutes (−0.59) are explained by relative expression and playing position, and the pre-specified sensitivity analysis demonstrates this rather than merely asserting it. Aerobic power expressed per kilogram of body mass, or, equivalently, running speed in a weight-bearing test, is systematically higher in smaller athletes. When the same aerobic result is expressed in absolute terms (L·min^−1^), the correlation with rebounds falls from −0.86 to −0.17 (*p* = 0.587), and the sole survivor of multiplicity control in this dataset ceases to exist (Figure 4b, Table S4). The coefficient was therefore a body-size artefact, and the size correction resolves it unambiguously. The direction is exactly what the literature predicts: guards attain significantly higher relative VO_2_max than centres in professional squads [11], guards and forwards display greater relative aerobic power than centres [67], and backcourt players attain higher VO_2_peak than frontcourt players on both laboratory and field tests [68]. Because rebounds and interior scoring are concentrated in the frontcourt [69], a negative association between relative aerobic capacity and these box-score categories is the arithmetic consequence of roster structure, not evidence that aerobic fitness impairs rebounding.

### 4.4. Anthropometry–Performance Associations Are Largely Explained by Playing Position

The remaining associations follow the same logic. Guards, being shorter, lighter and, on average, leaner, handle and distribute the ball and therefore accumulate assists, which accounts for the negative correlations of stature, body mass, Σ8 skinfolds and endomorphy with assists per game. Taller and heavier players operate closer to the basket and consequently take a shot portfolio dominated by short-range attempts, a distributional feature rather than a marksmanship advantage; league-wide analyses of the NBA over the past decade document a pronounced shift in shot selection towards the three-point line, with accuracy trends diverging between three-point and shorter-range attempts [70]. The positive correlations of BMI and mesomorphy with field-goal percentage would be expected if they reflect where a player shoots from rather than how well he shoots, and the mirror-image negative correlation with ectomorphy is the same phenomenon expressed through the linearity component. Three caveats apply, and they are severe enough to make this the most tentative part of the paper: the design cannot test the shot-location mechanism, since shot-distance data were not available; field-goal percentage rests on an unknown and, for low-minute players, certainly small number of attempts; and none of the four field-goal coefficients survives multiplicity control (*q* ≥ 0.078). They are offered as a hypothesis to be tested with shot-location data, not as a result.

Positional differences in box-score profiles are well documented and align with what was observed here. Across FIBA continental competitions in female players, centres record substantially more two-point makes and attempts and more offensive and defensive rebounds than guards [69]; the same positional signature emerges in external-load monitoring in male professional players [6,71] and in data-driven classifications that recover more than five functional profiles from competition statistics alone [39]. Our data reproduce that signature at the level of body build rather than of activity: the guard–big separation visible in Figure 2 maps onto the assists–rebounds separation visible in Figure 4, which is precisely what the confounding argument predicts. These patterns reflect positional specialisation rather than a direct causal influence of any single anthropometric variable on productivity, although the sex of some reference samples should be borne in mind when transferring them to a male roster.

The a priori hypothesis stated at the end of Section 1 is therefore supported, with one important qualification about what “supported” can mean in a design of this kind. Within a homogeneous elite squad, body size is so tightly bound to playing role that raw anthropometry–output correlations are confounded by position by construction: body size determines role, role determines statistical category, and the correlation between size and statistical category therefore carries little information about the causal contribution of body composition to performance. The evidence assembled here is consistent with that account on three independent grounds: the direction of every nominally significant coefficient corresponds to the known backcourt–frontcourt division of labour; the positional groups separate along both axes in every panel of Figure 4; and the one coefficient that survived multiplicity control was eliminated by a size correction, which is what a position-driven artefact should do and what a genuine physiological effect should not. It must nonetheless be stated plainly that the hypothesis was supported by convergent circumstantial evidence and not by a formal test. Formal separation would require position-adjusted or within-position estimation, which nine guards, six bigs and one wing/forward cannot support; Figure 4 shows that gradients are also visible within the guard group; and the correct verdict is therefore that playing position is a plausible common cause sufficient to explain the observed pattern, not a demonstrated one. Our findings are consistent with the modest and inconsistent results of the few studies that have attempted the same linkage: in National Collegiate Athletic Association (NCAA) women’s collegiate guards, no athletic performance test result was associated with game performance, and only fat-free mass correlated significantly with playing efficiency and rebounds per minute [40]. Profiling within a single team is valuable for individualised conditioning, load management and positional benchmarking, but it cannot, on its own, isolate the performance contribution of body composition. That question requires position-stratified or multi-team designs with adequate sample sizes, or longitudinal within-player designs in which each athlete serves as his own control.

### 4.5. Practical Applications

Practitioners can use a whole-roster ISAK and bioimpedance profile of this kind to benchmark players against positional norms, individualise nutrition and conditioning, and monitor body composition across a season, during which meaningful changes in anthropometric variables and somatotype do occur [21,25,31]. Adiposity in particular merits monitoring for reasons unrelated to box-score output: in a retrospective cohort of two male professional squads, higher preseason body-fat percentage was associated with a greater likelihood of high in-season injury burden, although the authors explicitly framed those findings as exploratory and hypothesis-generating [72]. Consensus recommendations [73], aligning with recent bibliometric trends in soccer [74], emphasise that body-composition assessment in sport should be organised around athlete health and availability rather than the pursuit of arbitrary compositional targets, and that observed compositional change should be interpreted against the measurement error of the technique employed [75]. Where bioimpedance is used, reporting raw parameters such as the phase angle alongside equation-derived estimates preserves information that is independent of the prediction model [27,51,52].

Equally important is what these data caution against. Coaches and analysts should not infer a player’s likely statistical contribution from body size within an elite squad, where size and role are inseparable; nor should within-team anthropometry–output correlations be used to justify individual selection, contract or playing-time decisions.

### 4.6. Limitations

Several limitations must be emphasised. First, the analysis is based on a single team with a small sample (14 players with paired anthropometric and game data, 12 for analyses involving aerobic performance), so statistical power is low, 80% power is reached only for *ρ* ≥ 0.69, and estimates are imprecise, as the wide confidence intervals show; BCa bootstrap intervals are themselves unstable at this sample size, which is why Fisher *z* intervals are reported alongside them and why the two are interpreted jointly. Second, 60 correlations were examined; the false discovery rate was controlled with the Benjamini–Hochberg procedure, and only one coefficient survived, so the remaining eleven nominally significant results are treated as exploratory throughout the manuscript, including in the Abstract. Third, the design is cross-sectional, precluding causal inference, and body size is structurally confounded with playing position; the small and uneven distribution of players across positional groups (guards *n* = 9; wings/forwards *n* = 1; bigs *n* = 6) prevented the position-adjusted analysis that would be required to address this confounding, so the position-based reading of the results is an interpretation supported by convergent evidence rather than a tested finding. Fourth, per-game values derive from a competition window of eight games, and field-goal percentage was taken from a source percentage field whose denominator could not be recovered, so no minimum-attempt criterion could be applied; percentages ranged from 31% to 82%, and the four associations involving this variable are correspondingly fragile. Fifth, aerobic capacity was assessed with a field test rather than by gas analysis; the maximal aerobic speed is a measured quantity, but the VO_2_max derived from it is implausible in absolute terms for this population, so the variable is used only as an ordinal ranking, and the composite variable omits steals, blocks and turnovers, which were unavailable, while box-score metrics in general capture offensive volume and accuracy but not defensive contribution, spacing or other off-ball value. Sixth, the bioimpedance fat/fat-free partition failed a formal coherence audit (Section 2.4), implied fat mass was zero or negative in 5 of 16 players and BMI did not equal FMI + FFMI in 8 of 12, so fat mass, percentage body fat, FMI and FFMI were removed from the study altogether and adiposity is characterised solely by Σ8 skinfolds and endomorphy; the phase angle was retained because it is a raw parameter, but it came from the same export and the same session, and an instrument-related contribution to its unusually high value cannot be excluded. Seventh, several per-game fields in the source dataset had been altered by spreadsheet date formatting; per-game values were therefore recomputed from season totals verified against the official portal, but this required discarding the affected cells. Eighth, assessments were made at a single time point, three days before the first game, so seasonal change in body composition could not be described, and no measure of training load, nutritional intake or injury status was available. Finally, because the club is identified, individual data cannot be considered fully anonymous, which constrains the granularity at which individual results can be reported. Each of these limitations argues for cautious, descriptive interpretation.

## 5. Conclusions

In this professional basketball roster competing at continental level, the anthropometric profile was characteristic of the elite game—tall (194.6 ± 9.1 cm), long-limbed (arm span exceeding stature by 4.8 cm), lean (Σ8 82 ± 29 mm) and balanced-mesomorphic (2.5–4.5–3.0), and, in the Mexican context, notably less endomorphic than national athletic reference profiles. This descriptive profile is the study’s substantive contribution and provides positional benchmarks for Latin American professional basketball, where such data are scarce. On the a priori hypothesis, the verdict is explicit: it was supported, but by convergent circumstantial evidence rather than by a formal test. Of 60 exploratory correlations, only one survived false-discovery-rate control, and a pre-specified size correction showed that this single survivor was an artefact of expressing aerobic capacity per unit of body mass rather than a physiological association; the remaining eleven nominally significant coefficients were imprecise, did not survive multiplicity control, and all pointed in the directions predicted by the backcourt–frontcourt division of labour. No association can therefore be attributed to a direct effect of body size or composition on game output, and the confounding by playing position could not itself be estimated in a single squad of this size. The practical implication is unambiguous: whole-roster kinanthropometric profiling is valuable for individualised conditioning, nutrition and positional benchmarking, but within-team anthropometry–output correlations should not be used to justify selection, contract or playing-time decisions. The question of whether body composition contributes to competitive output requires position-stratified or multi-team designs with adequate sample sizes, or longitudinal within-player designs in which each athlete serves as his own control.

## Figures and Tables

**Figure 1:**
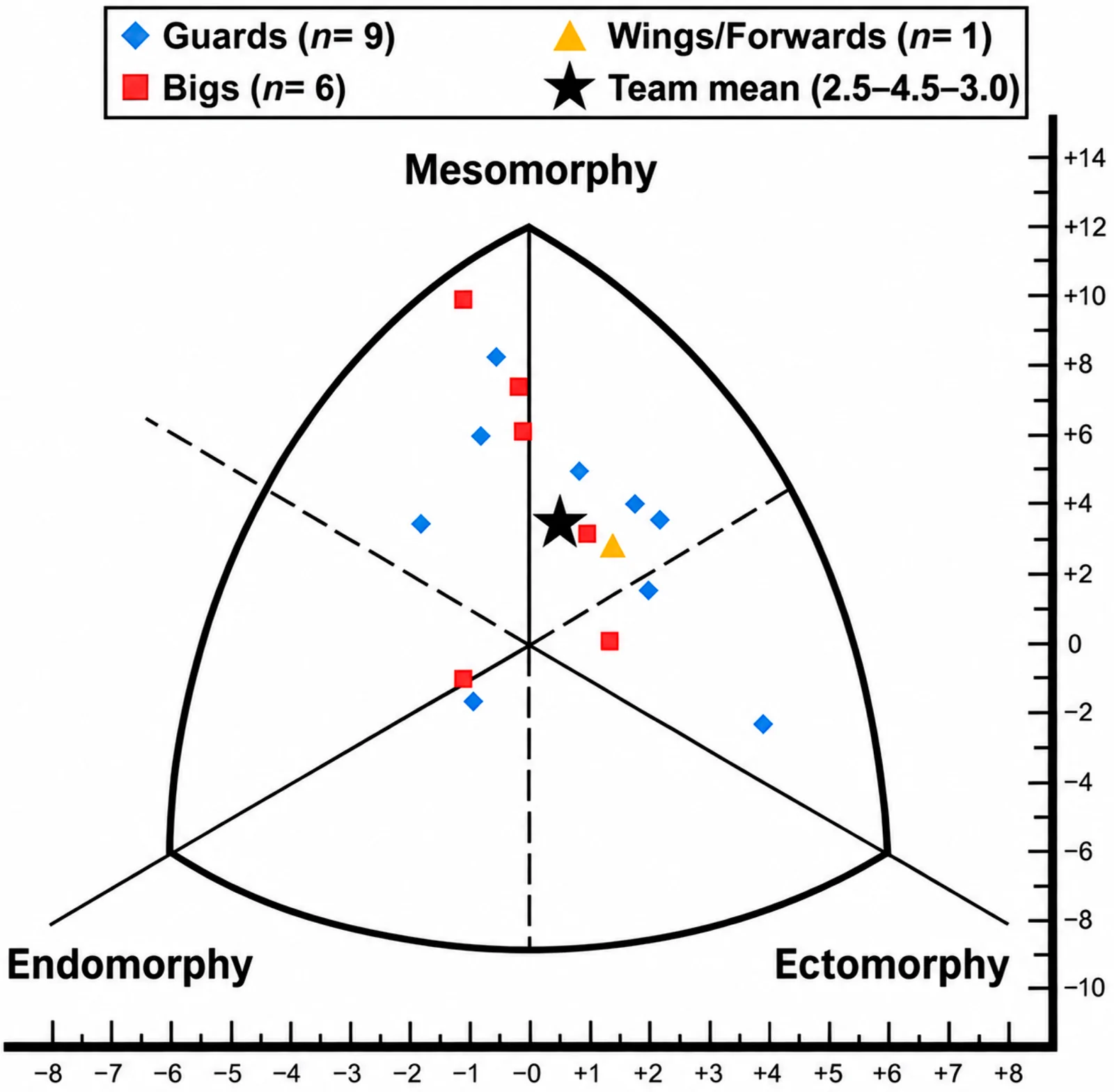
Heath–Carter somatochart of the roster (*n* = 16). Each marker represents one player’s somatotype, coded by positional group (guards, *n* = 9; wings/forwards, *n* = 1; bigs, *n* = 6); the star denotes the team mean (2.5–4.5–3.0, balanced mesomorph). Axes follow the standard somatochart projection: X = ectomorphy − endomorphy, where positive values indicate greater linearity relative to adiposity; Y = 2·mesomorphy − (endomorphy + ectomorphy), where positive values indicate musculoskeletal robustness dominating the other two components. The dispersion of individual points reflects the heterogeneity of body builds coexisting within a single professional squad.

**Figure 2:**
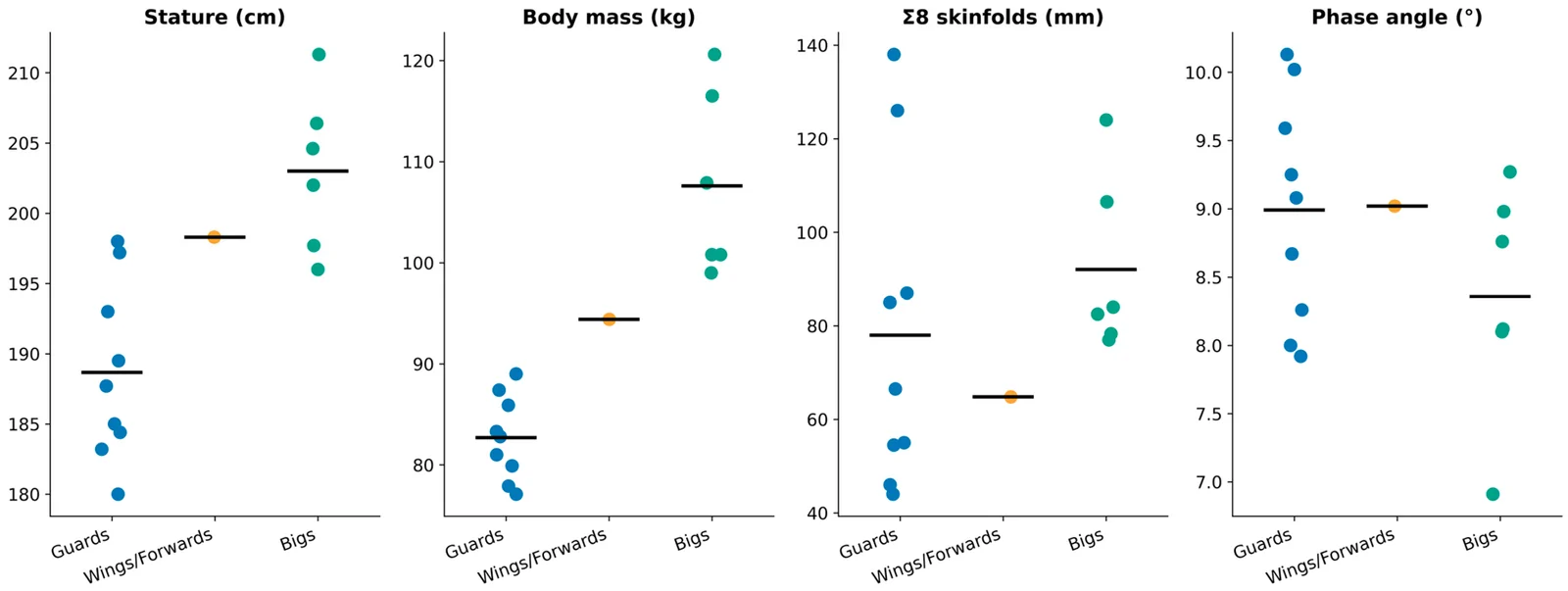
Distribution of stature (cm), body mass (kg), the sum of eight ISAK skinfolds (Σ8, mm) and phase angle (°) across positional groups (guards, *n* = 9; wings/forwards, *n* = 1; bigs, *n* = 6). Each point is an individual player; horizontal bars denote group means. Stature and body mass separate clearly between guards and bigs, whereas Σ8 skinfolds overlap, with the two highest individual values recorded in guards, and phase angle overlaps almost completely. Phase angle, a raw bioelectrical parameter independent of the fat/fat-free partition (which failed the data-quality audit reported in Section 2.4), is displayed. Because the wing/forward group comprises a single player, group means are descriptive and no statistical comparison was performed.

**Figure 3:**
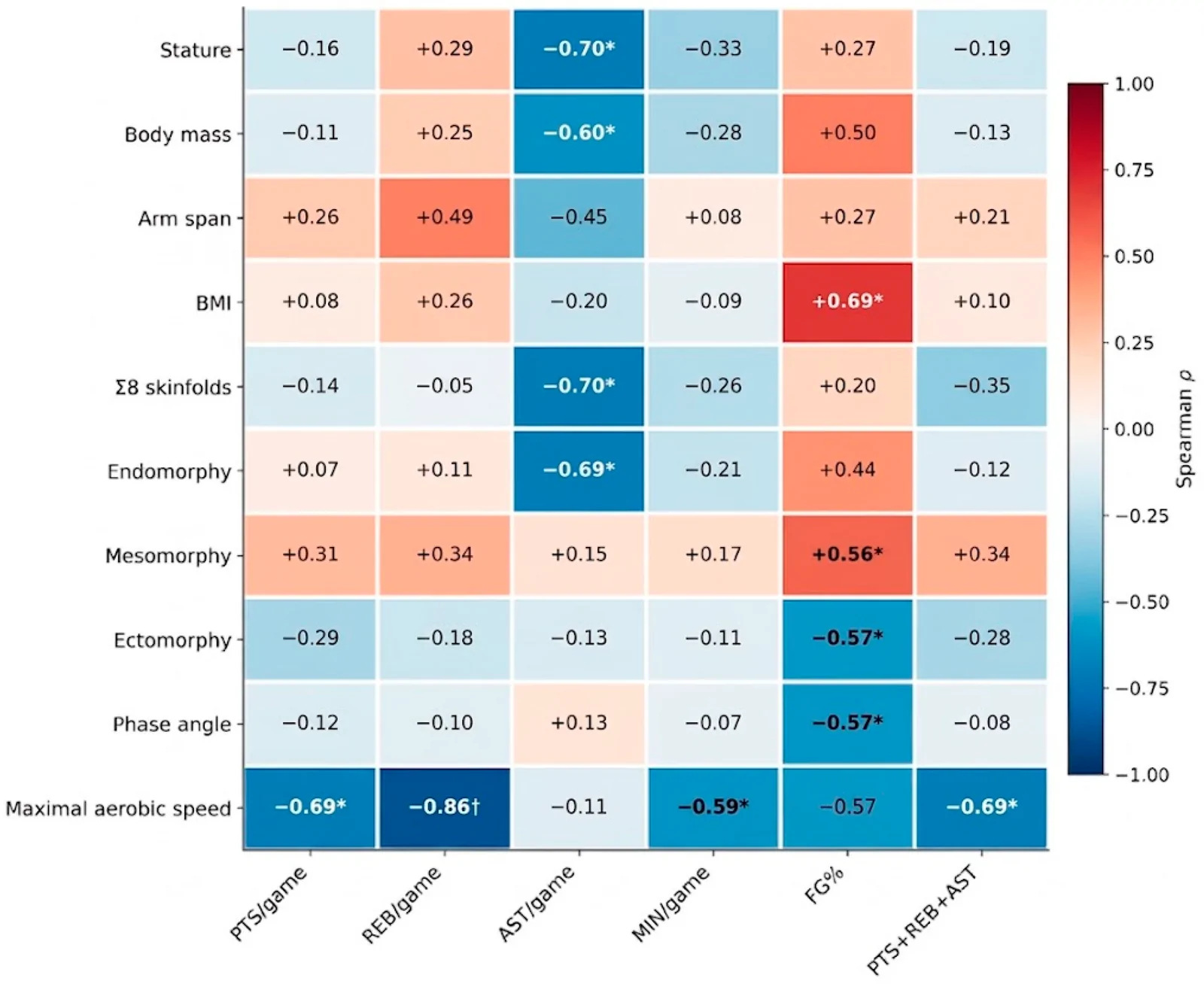
Spearman rank correlation matrix between anthropometric and body-composition descriptors (rows) and per-game output variables (columns), computed on the 14 players with paired data (12 for the row involving maximal aerobic speed). Cell colour encodes *ρ* across the full range −1 to +1, with negative and positive associations rendered in opposing hues and values near zero rendered neutral. Asterisks (*) denote nominal *p* < 0.05 in an exploratory analysis; the dagger (†) denotes the single coefficient that survives Benjamini–Hochberg false-discovery-rate control at *q* < 0.05. The marked cells must not be read as firm findings: as Table 2 shows, their confidence intervals are wide; several extend to the boundary of the parameter space, two include zero under one of the two interval methods, and eleven of the twelve do not survive multiplicity control. Fat-derived bioimpedance variables (fat-mass index, fat-free-mass index and percentage body fat) are omitted for the reason given in Section 2.4.

**Figure 4:**
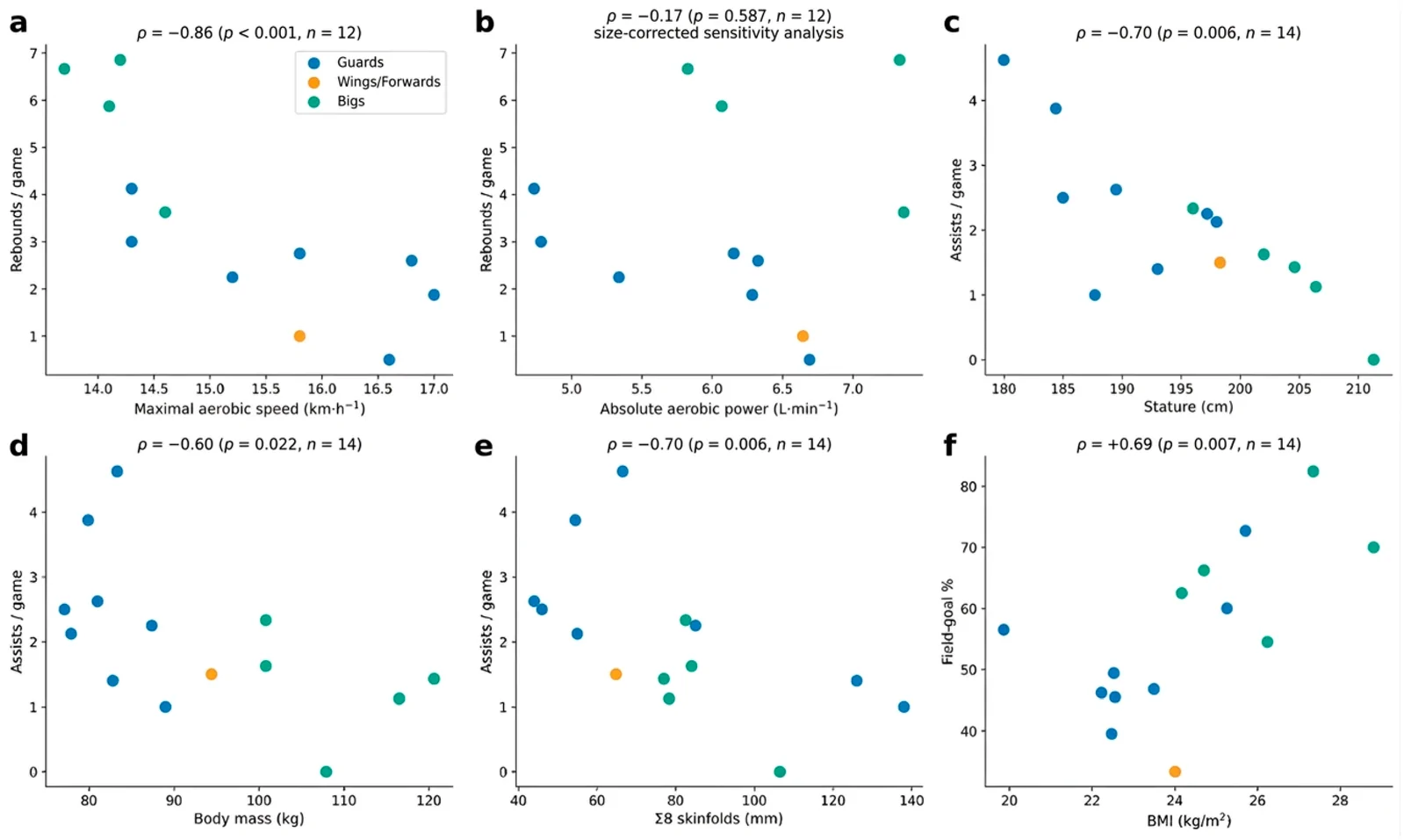
Selected anthropometry–performance associations, with each player coded by positional group. Panels show maximal aerobic speed versus rebounds per game (**a**); the same outcome against absolute aerobic power (L·min^−1^), as the size-corrected sensitivity analysis (**b**); stature (**c**), body mass (**d**) and Σ8 skinfolds (**e**) versus assists per game; and BMI versus field-goal percentage (**f**). In panels (**a**) and (**c**–**f**), the positional groups separate along both axes and therefore contribute substantially to the overall association, although within-group gradients are also visible, particularly among guards, so the pooled coefficients cannot be attributed to between-group separation alone. Panel (**b**) shows that the strongest association in the dataset is not recovered once aerobic power is expressed independently of body mass. Group sizes (guards *n* = 9; bigs *n* = 6; wings/forwards *n* = 1) preclude formal position-adjusted estimation; the figure is presented to make the confounding between body size and playing role visible, not to quantify it.

**Table 1:** Anthropometric, body-composition and per-game performance characteristics of the team (mean ± SD, median [IQR], range and *n*).

Variable	Mean ± SD	Median [IQR]	Range	*n*
Age (years)	30.8 ± 4.5	30.5 [28.5–33.0]	23–40	16
Body mass (kg)	92.8 ± 13.6	88.2 [82.4–100.8]	77.1–120.6	16
Stature (cm)	194.6 ± 9.1	196.6 [187.0–199.2]	180–211	16
Arm span (cm)	199.4 ± 12.3	197.4 [191.4–206.7]	179–227	16
BMI (kg/m^2^)	24.4 ± 2.2	24.4 [22.6–25.6]	19.9–28.8	16
Σ8 skinfolds (mm)	82 ± 29	80.4 [62.4–91.9]	44–138	16
Endomorphy	2.5 ± 1.0	2.3 [2.0–2.7]	1.3–4.8	16
Mesomorphy	4.5 ± 1.3	4.4 [3.7–5.3]	2.2–7.3	16
Ectomorphy	3.0 ± 1.0	3.0 [2.3–3.6]	1.6–5.4	16
Phase angle (°)	8.8 ± 0.8	8.9 [8.1–9.3]	6.9–10.1	16
Maximal aerobic speed (km·h^−1^)	15.3 ± 1.2	15.2 [14.3–16.6]	13.7–17.0	13
Estimated VO_2_max (mL·kg^−1^·min^−1^)	67.4 ± 7.1	66.8 [61.4–75.2]	57.8–77.6	13
Points per game	8.8 ± 5.6	6.3 [4.1–14.3]	2.9–19.2	16
Rebounds per game	4.1 ± 3.3	2.9 [2.2–5.6]	0.5–14.0	16
Assists per game	2.0 ± 1.2	1.9 [1.3–2.5]	0.0–4.6	16
Minutes per game	17.5 ± 6.9	17.3 [11.2–21.5]	7.5–30.0	16
PTS + REB + AST per game	14.9 ± 8.0	13.3 [8.6–22.6]	5.0–29.0	16
Field-goal %	53.6 ± 14.6	52.0 [44.6–63.4]	31–82	16

Note: BMI, body mass index; Σ8, sum of eight ISAK skinfolds; IQR, interquartile range; SD, standard deviation; PTS + REB + AST, combined points, rebounds and assists per game; maximal aerobic speed, running speed corresponding to the last stage completed in the 20 m multistage shuttle run. *n* denotes the number of players with available data for each variable (anthropometric *n* = 16 assessed players; performance *n* = 16 players with competition minutes; these sets overlap but are not identical; see Figure S1). Median [IQR] is reported alongside mean ± SD because several per-game distributions are skewed. Estimated VO_2_max is a strictly increasing linear function of maximal aerobic speed (VO_2_max = 6.0 × speed − 24.4) and is given for descriptive comparison only; it is interpreted solely as a within-squad ordinal ranking and never as an absolute physiological value (Section 2.4 and Section 4.3). Fat-mass index, fat-free-mass index and percentage body fat are not reported because the bioimpedance fat/fat-free partition failed the data-quality audit described in Section 2.4.

**Table 2:** Spearman associations between anthropometric/body-composition variables and per-game output reaching nominal *p* < 0.05, with bias-corrected and accelerated (BCa) bootstrap 95% confidence intervals (2000 resamples), Fisher *z* 95% confidence intervals computed with the Bonett–Wright standard error, exact *p* values and Benjamini–Hochberg *q* values computed across the full set of 60 tests. Twelve of the 60 tested correlations are shown; only the first survives false-discovery-rate control at *q* < 0.05. This table summarises the nominally significant subset and is not a list of established findings; the complete matrix of 60 coefficients, with intervals and exact *p* and *q* values, is given in Table S5.

Association	*ρ*	BCa 95% CI	Fisher *z* 95% CI	*p*	*q*	*n*
Maximal aerobic speed × rebounds/game	−0.86	−0.98, −0.57	−0.97, −0.49	<0.001	0.018	12
Stature × assists/game	−0.70	−1.00, +0.01	−0.91, −0.20	0.006	0.078	14
Σ8 skinfolds × assists/game	−0.70	−0.88, −0.32	−0.91, −0.20	0.006	0.078	14
Maximal aerobic speed × PTS + REB + AST/game	−0.69	−0.94, −0.22	−0.92, −0.13	0.012	0.110	12
Maximal aerobic speed × points/game	−0.69	−0.93, −0.19	−0.92, −0.12	0.013	0.110	12
BMI × field-goal %	+0.69	+0.19, +0.89	+0.18, +0.91	0.007	0.078	14
Endomorphy × assists/game	−0.69	−0.88, −0.32	−0.91, −0.18	0.007	0.078	14
Body mass × assists/game	−0.60	−0.82, −0.21	−0.87, −0.05	0.022	0.169	14
Maximal aerobic speed × minutes/game	−0.59	−0.88, −0.10	−0.88, +0.04	0.045	0.226	12
Phase angle × field-goal %	−0.57	−0.83, −0.13	−0.86, −0.02	0.032	0.202	14
Ectomorphy × field-goal %	−0.57	−0.85, −0.05	−0.86, −0.01	0.034	0.202	14
Mesomorphy × field-goal %	+0.56	−0.03, +0.87	−0.01, +0.85	0.039	0.212	14

Note: *ρ*, Spearman rank correlation coefficient; CI, confidence interval; BCa, bias-corrected and accelerated bootstrap; Fisher *z*, interval from the Fisher *z* transformation with the Bonett–Wright standard error; *q*, Benjamini–Hochberg false-discovery-rate-adjusted *p* value across all 60 tests; BMI, body mass index; Σ8, sum of eight ISAK skinfolds; PTS + REB + AST, combined points, rebounds and assists per game. Maximal aerobic speed is the running speed at the last stage completed in the 20 m multistage shuttle run; the coefficients are numerically identical to those obtained with the derived VO_2_max estimate, which is a strictly increasing function of that speed. Intervals whose bounds approach or cross zero indicate very low precision; the two interval types disagree for two coefficients, which are consequently the least stable estimates in the table. The four associations involving field-goal percentage rest on an unknown number of shot attempts (Section 2.5) and are the least reliable in this analysis.

## Data Availability

The de-identified dataset is available from the corresponding author on reasonable request. Official competition statistics are publicly available from the Basketball Champions League Americas (https://www.fiba.basketball/es/events/basketball-champions-league-americas-25-26/teams/astros-de-jalisco#statistics; accessed on 20 February 2026).

## Supplementary Materials

The following supporting information can be downloaded at https://www.mdpi.com/article/10.3390/jfmk11040386/s1: Figure S1, Participant flow; Table S1, STROBE (Strengthening the Reporting of Observational Studies in Epidemiology) Checklist for Cross-Sectional Studies; Table S2, Sensitivity analysis excluding the single wing/forward: Spearman’s rank correlation (ρ) matrix between anthropometric/body-composition variables and per-game performance metrics; Table S3, Coherence check and implausible values flagged in baseline body-composition and anthropometric assessments; Table S4, Correlations between aerobic fitness (relative and absolute), partial correlations controlling for body mass, and per-game performance metrics; Table S5, Complete Spearman correlation (ρ) matrix between anthropometric/body-composition variables and per-game performance variables; Table S6, Descriptive anthropometric, body-composition and per-game characteristics by positional group.

## Abbreviations

The following abbreviations are used in this manuscript:

AST	Assists
BCa	Bias-corrected and accelerated
BCLA	Basketball Champions League Americas
BIA	Bioelectrical impedance analysis
BMI	Body mass index
CI	Confidence interval
FDR	False discovery rate
FFMI	Fat-free-mass index
FG%	Field-goal percentage
FIBA	International Basketball Federation
FMI	Fat-mass index
IQR	Interquartile range
ISAK	International Society for the Advancement of Kinanthropometry
NBA	National Basketball Association
NCAA	National Collegiate Athletic Association
PTS	Points
REB	Rebounds
SD	Standard deviation
STROBE	Strengthening the Reporting of Observational Studies in Epidemiology
Σ8	Sum of eight ISAK skinfolds
VO_2_max	Maximal oxygen uptake
