# Supplementary material for: Anthropometric Profile, Body Composition, and Game Performance of the Mexican Professional Basketball League Champion Team During the 2025–2026 Basketball Champions League Americas: A Cross-Sectional Study
Source: J Funct Morphol Kinesiol. 2026 Sep 26;11(4):386. doi: 10.3390/jfmk11040386 (PMC13692546; doi:10.3390/jfmk11040386)
Supplement: Supplementary file 1 [file jfmk-11-00386-s001.zip › jfmk-4566995-supplementary.pdf]

Supplementary material

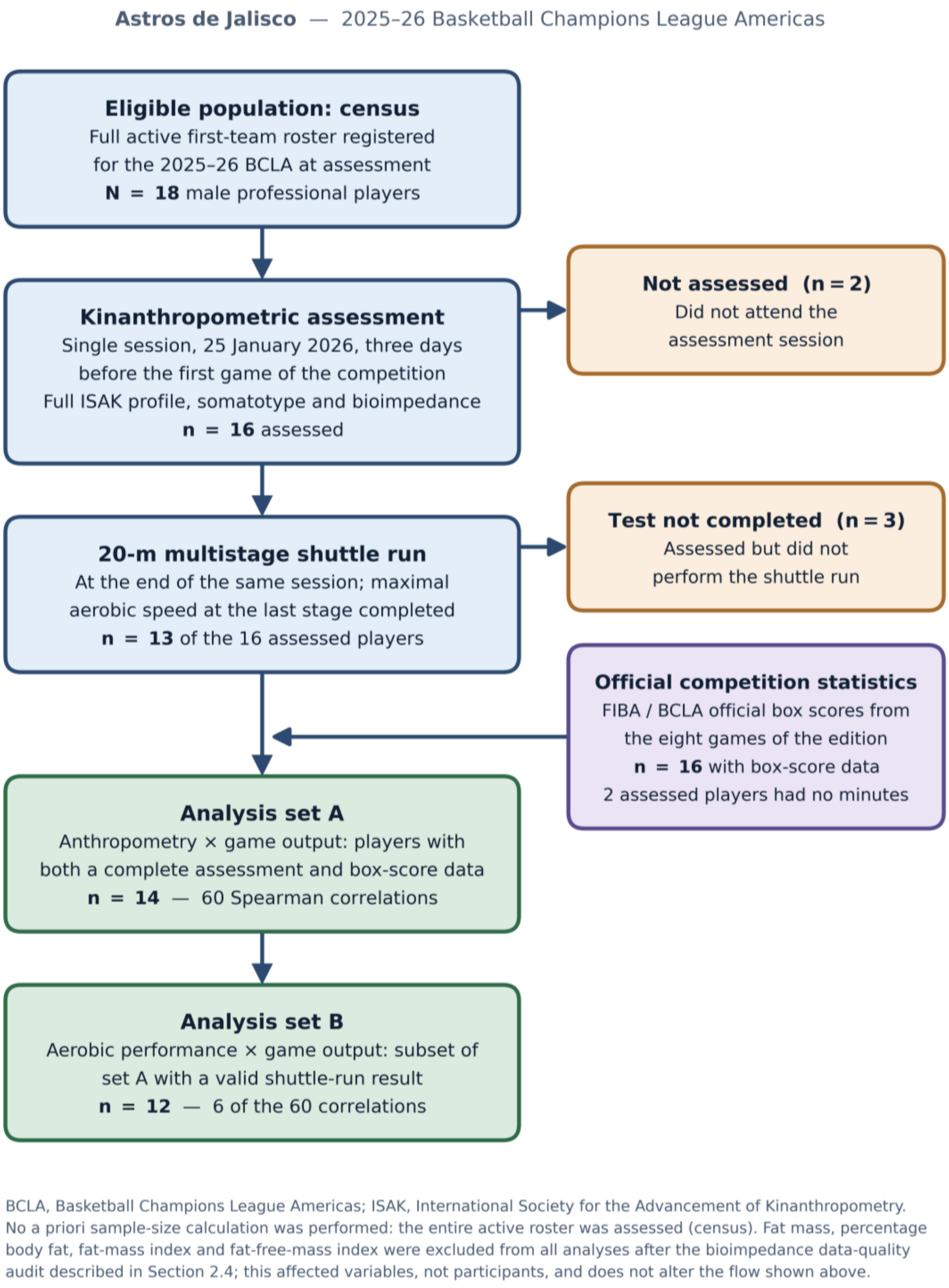

Figure S1. Participant flow.

Table S1. STROBE (Strengthening the Reporting of Observational Studies in Epidemiology) Checklist for Cross-Sectional Studies.

Item No.	Section/Topic	STROBE Recommendation	Reported on Page/Section
**1a**	**Title**	Indicate the study's design with a commonly used term in the title or the abstract.	**Title/Page 1:** Expressly stated as *"...A Cross-Sectional Study"*.
**1b**	**Abstract**	Provide in the abstract an informative and balanced summary of what was done and what was found.	**Abstract/Page 1:** Summarises background, objectives, methods, main results, and conclusions.
**2**	**Background/Rationale**	Explain the scientific background and rationale for the investigation being reported.	**Section 1 (Introduction):** Paragraphs 1–4 (Pages 1–2).
**3**	**Objectives**	State specific objectives, including any prespecified hypotheses.	**Section 1 (Introduction):** Paragraph 5 (Page 2). Details the two objectives and the *a priori* hypothesis.
**4**	**Study design**	Present key elements of study design early in the paper.	**Section 2.1 (Study Design):** Paragraph 1 (Page 2). Observational, cross-sectional, single-team study.
**5**	**Setting**	Describe the setting, locations, and relevant dates, including periods of recruitment, exposure, follow-up, and data collection.	**Sections 2.1 and 2.2 (Setting and Ethical Approval):** Page 2. Single-day assessment on 25 January 2026 in Guadalajara, Mexico; competition window comprising 8 games in the 2025–26 BCLA.
**6a**	**Participants**	Give the eligibility criteria, and the sources and methods of selection of participants.	**Section 2.3 (Participants):** Page 2. Complete census of the active roster (N = 18 registered; 16 assessed; 13 completed shuttle run; 14 paired anthropometry–box-score; 12 paired for aerobic analyses).
**6b**	**Participants**	For matched studies, give matching criteria and number of exposed and unexposed.	**Not applicable** (cross-sectional census design with no control group or matching).
**7**	**Variables**	Clearly define all outcomes, exposures, predictors, potential confounders, and effect modifiers. Give diagnostic criteria, if applicable.	**Sections 2.4 and 2.5 (Anthropometry/Game Statistics):** Pages 2–3. Anthropometric variables (ISAK), somatotype, phase angle (BIA), maximal aerobic speed, and per-game metrics (*PTS, REB, AST, MIN, FG%*).
**8**	**Data sources/Measurement**	For each variable of interest, give sources of data and	**Sections 2.4 and 2.5:** Pages 2–3. ISAK Level 2 protocol, BIA *seca mBCA 525*, 20-m multistage

Item No.	Section/Topic	STROBE Recommendation	Reported on Page/Section
		details of methods of assessment (measurement).	shuttle run, and official FIBA/BCLA box-score statistics.
9	**Bias**	Describe any efforts to address potential sources of bias.	**Sections 2.4 and 2.5:** Single ISAK Level 2 accredited measurer (eliminating inter-observer error); BIA data-quality audit (removal of unreliable fat-mass data); independent cross-checking of box-score data to correct spreadsheet formatting faults.
10	**Study size**	Explain how the study size was arrived at.	**Section 2.3 (Participants):** Page 2, Paragraph 2. Explanation of the census approach (entire available roster) and transparent report of the resulting precision/power for $N = 14$ and $N = 12$.
11	**Quantitative variables**	Explain how quantitative variables were handled in the analyses. If applicable, describe which groupings were chosen and why.	**Sections 2.4, 2.5 and 2.6:** Continuous variables reported as mean ± SD and median [IQR]; use of directly measured maximal aerobic speed instead of estimated VO2max for inferential analyses.
12a	**Statistical methods**	Describe all statistical methods, including those used to control for confounding.	**Section 2.6 (Statistical Analysis):** Page 3. Spearman's rank correlation coefficient (ρ), BCa bootstrap and Fisher z 95% CIs.*ρz*
12b	**Statistical methods**	Describe any methods used to examine subgroups and interactions.	**Sections 2.3 and 3.3:** Descriptive profiling by positional groups (guards, wings/forwards, bigs); no inferential testing between subgroups performed due to small sample size.
12c	**Statistical methods**	Explain how missing data were addressed.	**Section 2.6:** Complete-case analysis; no imputation was performed.
12d	**Statistical methods**	If applicable, describe analytical methods taking account of sampling strategy.	**Sections 2.6 and 3.4:** False discovery rate (FDR) controlled using the Benjamini–Hochberg procedure (q-values) across the 60 exploratory correlations.*q*
12e	**Statistical methods**	Describe any sensitivity analyses.	**Sections 2.6 and 3.4:** Pages 3 and 5. Two pre-specified analyses: (i) excluding the single wing/forward *(Table S2),* and (ii) body-size correction for the aerobic variable *(Table S4).*
13a	**Participants**	Report numbers of individuals at each stage of study.	**Section 2.3 and Figure S1:** 18 rostered; 16 assessed; 13 completed shuttle run; 14 paired anthropometry–box-score; 12 paired for aerobic analyses.

Item No.	Section/Topic	STROBE Recommendation	Reported on Page/Section
**13b**	**Participants**	Give reasons for non-participation at each stage.	**Section 2.3:** Page 2, Paragraph 3. Two players did not attend the assessment; two assessed players recorded no competition minutes.
**13c**	**Participants**	Consider use of a flow diagram.	**Supplementary Materials:** Included as **Figure S1**(*Participant flow diagram*).
**14a**	**Descriptive data**	Give characteristics of study participants (e.g. demographic, clinical, social) and information on exposures and potential confounders.	Section 3.1, Table 1, and Table S6: Pages 3–4. Age, body mass, stature, arm span, BMI, 8 skinfolds, somatotype, and phase angle.Σ
**14b**	**Descriptive data**	Indicate number of participants with missing data for each variable of interest.	**Table 1:** The exact number of players (n) contributing data is specified for each variable.
**15**	**Outcome data**	Report numbers of outcome events or summary measures.	Section 3.1, Table 1, and Table S6: Summary of points, rebounds, assists, minutes, FG%, and combined PTS+REB+AST per game.
**16a**	**Main results**	Give unadjusted estimates and, if applicable, confounder-adjusted estimates and their precision (e.g., 95% confidence interval).	**Section 3.4, Table 2, Figure 3, and Figure 4:** Pages 4–5. Comprehensive mapping of 60 correlations providing ρ coefficients, BCa bootstrap CIs, Fisher z CIs, exact p-values, and FDR q-values.ρz
**16b**	**Main results**	Report category boundaries when continuous variables were categorized.	**Not applicable:** All quantitative variables were modelled continuously.
**17**	**Other analyses**	Report other analyses done—e.g. analyses of subgroups and interactions, and sensitivity analyses.	Section 3.4, Table S2, and Table S4: Page 5. Sensitivity analysis omitting the single forward (Table S2), and size-adjustment sensitivity analysis for the aerobic capacity variable (Table S4).
**18**	**Key results**	Summarise key results with reference to study objectives.	**Section 4 (Discussion):** Page 5, Paragraph 1. Summary of primary findings tied directly to the objectives and hypotheses.
**19**	**Limitations**	Discuss limitations of the study, taking into account sources of potential bias or imprecision.	**Section 4.6 (Limitations):** Page 7. Detailed discussion of 8 key limitations (sample size, CI imprecision, multiplicity, structural positional confounding, BIA algorithm failure, etc.).
**20**	**Interpretation**	Give a cautious overall interpretation of results considering objectives, limitations,	**Sections 4.1–4.4:** Pages 5–7. Cautious framing addressing structural confounding by playing position and critical review of BIA parameters.

Item No.	Section/Topic	STROBE Recommendation	Reported on Page/Section
		multiplicity of analyses, and other relevant evidence.	
**21**	**Generalisability**	Discuss the generalisability (external validity) of the study results.	**Sections 4.1, 4.4, and 4.5:** Pages 5–7. Applicability limited to the Latin American professional basketball context and constraints on individual-level extrapolation.
**22**	**Funding**	Give the source of funding and the role of the funders for the present study.	**Funding Section/Page 8:** *"This research received no external funding."* Explicit declaration of no external funding and independence from the sports club.

Table S2. Sensitivity analysis excluding the single wing/forward: Spearman's rank correlation (ρ) matrix between anthropometric/body-composition variables and per-game performance metrics (n = 13; n = 11 for maximal aerobic speed).

Anthropometric Variable	Performance Metric	ρ	BCa Lower	BCa Upper	Fisher's z Lower	Fisher's z Upper	p-value	n	q-value (BH)
Stature	PTS/game	-0.137	-0.741	0.56	-0.642	0.45	0.6545	13	0.8727
	REB/game	0.377	-0.358	0.81	-0.24	0.777	0.2043	13	0.596
	AST/game	-0.709	-0.994	0.141	-0.918	-0.189	0.0067	13	0.0804
	MIN/game	-0.286	-0.788	0.395	-0.729	0.326	0.3433	13	0.6645
	FG%	0.357	-0.497	0.799	-0.26	0.767	0.2309	13	0.6165
	PTS+REB+AST	-0.148	-0.745	0.518	-0.648	0.441	0.6286	13	0.8727
Body mass	PTS/game	-0.121	-0.712	0.626	-0.631	0.462	0.6936	13	0.8854
	REB/game	0.292	-0.418	0.794	-0.32	0.732	0.333	13	0.6645
	AST/game	-0.597	-0.834	-0.17	-0.877	-0.016	0.0312	13	0.1811
	MIN/game	-0.292	-0.757	0.37	-0.732	0.32	0.333	13	0.6645
	FG%	0.605	0.136	0.881	0.027	0.88	0.0284	13	0.1811
	PTS+REB+AST	-0.113	-0.697	0.612	-0.626	0.469	0.7137	13	0.8921
Arm span	PTS/game	0.346	-0.363	0.843	-0.27	0.761	0.2466	13	0.6165
	REB/game	0.591	-0.006	0.886	0.008	0.874	0.0332	13	0.1811
	AST/game	-0.44	-0.859	0.316	-0.808	0.176	0.1329	13	0.4984
	MIN/game	0.173	-0.482	0.715	-0.421	0.664	0.5712	13	0.8727
	FG%	0.352	-0.566	0.849	-0.265	0.764	0.2387	13	0.6165
	PTS+REB+AST	0.308	-0.348	0.812	-0.306	0.741	0.3064	13	0.6645
BMI	PTS/game	0.082	-0.421	0.685	-0.492	0.607	0.789	13	0.9104
	REB/game	0.292	-0.39	0.788	-0.321	0.732	0.3337	13	0.6645
	AST/game	-0.154	-0.581	0.426	-0.652	0.437	0.6158	13	0.8727
	MIN/game	-0.091	-0.613	0.458	-0.612	0.485	0.768	13	0.9104
	FG%	0.731	0.238	0.91	0.229	0.926	0.0045	13	0.0675
	PTS+REB+AST	0.137	-0.421	0.746	-0.45	0.642	0.6545	13	0.8727
Σ8 skinfolds	PTS/game	-0.269	-0.775	0.591	-0.72	0.341	0.3737	13	0.698
	REB/game	-0.127	-0.71	0.584	-0.635	0.458	0.6804	13	0.8854
	AST/game	-0.742	-0.941	-0.435	-0.929	-0.249	0.0037	13	0.0675
	MIN/game	-0.382	-0.843	0.238	-0.78	0.235	0.1972	13	0.596
	FG%	0.104	-0.508	0.721	-0.475	0.621	0.7343	13	0.8991
	PTS+REB+AST	-0.412	-0.853	0.301	-0.795	0.205	0.1618	13	0.5711

Endomorphy	PTS/game	-0.071	-0.636	0.63	-0.599	0.5	0.8166	13	0.9245
	REB/game	0.044	-0.548	0.707	-0.52	0.581	0.8865	13	0.9464
	AST/game	-0.736	-0.912	-0.377	-0.928	-0.239	0.0041	13	0.0675
	MIN/game	-0.396	-0.771	0.283	-0.787	0.221	0.1802	13	0.596
	FG%	0.374	-0.427	0.721	-0.243	0.775	0.2086	13	0.596
	PTS+REB+AST	-0.198	-0.706	0.528	-0.678	0.401	0.5171	13	0.8618
Mesomorphy	PTS/game	0.264	-0.32	0.734	-0.346	0.717	0.3839	13	0.698
	REB/game	0.333	-0.295	0.775	-0.283	0.754	0.2664	13	0.6394
	AST/game	0.137	-0.476	0.72	-0.45	0.642	0.6545	13	0.8727
	MIN/game	0.088	-0.49	0.614	-0.487	0.61	0.7749	13	0.9104
	FG%	0.555	-0.082	0.859	-0.04	0.859	0.049	13	0.2262
	PTS+REB+AST	0.302	-0.297	0.812	-0.311	0.738	0.3156	13	0.6645
Ectomorphy	PTS/game	-0.231	-0.69	0.358	-0.698	0.374	0.4481	13	0.7682
	REB/game	-0.151	-0.727	0.513	-0.65	0.439	0.6217	13	0.8727
	AST/game	-0.143	-0.718	0.486	-0.645	0.446	0.6415	13	0.8727
	MIN/game	-0.033	-0.54	0.535	-0.574	0.528	0.9147	13	0.9464
	FG%	-0.582	-0.893	-0.043	-0.871	0.004	0.0367	13	0.1835
	PTS+REB+AST	-0.253	-0.743	0.399	-0.71	0.355	0.4048	13	0.7144
Phase angle	PTS/game	-0.033	-0.703	0.611	-0.574	0.528	0.9149	13	0.9464
	REB/game	-0.019	-0.587	0.634	-0.564	0.537	0.9502	13	0.9663
	AST/game	0.165	-0.569	0.762	-0.428	0.659	0.5905	13	0.8727
	MIN/game	0.006	-0.553	0.573	-0.547	0.555	0.9858	13	0.9858
	FG%	-0.604	-0.881	-0.036	-0.88	-0.026	0.0287	13	0.1811
	PTS+REB+AST	-0.033	-0.574	0.628	-0.574	0.528	0.9149	13	0.9464
Maximal aerobic speed	PTS/game	-0.656	-0.941	0.022	-0.914	-0.022	0.0284	11	0.1811
	REB/game	-0.893	-1	-0.708	-0.978	-0.549	0.0002	11	0.012
	AST/game	-0.041	-0.648	0.647	-0.626	0.573	0.9047	11	0.9464
	MIN/game	-0.565	-0.905	0.093	-0.882	0.106	0.0702	11	0.2808
	FG%	-0.565	-0.9	0.086	-0.882	0.106	0.0702	11	0.2808
	PTS+REB+AST	-0.679	-0.937	-0.007	-0.921	-0.058	0.0216	11	0.1811

Notes: ρ = Spearman's rank correlation coefficient. BCa = Bias-corrected and accelerated bootstrap 95% Confidence Interval. q_BH = Benjamini-Hochberg false discovery rate adjusted p-value. Bold values indicate statistically significant correlations ($p < 0.05$ or $q < 0.05$).

Table S3. Coherence check and implausible values flagged in baseline body-composition and anthropometric assessments.

ID	Mass (kg)	Stature (cm)	BMI	FFM (kg)	FFMI	FMI	PhA (°)	Implied FM (kg)	Implied BF (%)	Δ (BMI-FMI-FFMI)	Flag
1	77.1	185.0	22.53	72.86	21.95	1.28	10.02	4.24	5.50	-0.70	Implausible: <8% fat
2	79.9	184.4	23.50	74.04	21.77	1.72	8.26	5.86	7.33	0.01	Implausible: <8% fat
3	99.0	197.7	25.33	84.45	21.61	3.72	9.27	14.55	14.70	0.00	Coherence check
4	100.8	202.0	24.70	77.47	20.29	6.11	8.10	23.33	23.14	-1.70	Coherence check
5	77.9	198.0	19.87	85.56	22.41	0.87	9.25	-7.66	-9.83	-3.41	Implausible: FM ≤ 0
6	89.0	187.7	25.26	75.29	21.53	3.92	9.08	13.71	15.40	-0.19	Coherence check
7	120.6	204.6	28.81	101.97	24.36	4.45	8.76	18.63	15.45	0.00	Coherence check
8	82.8	193.0	22.23	80.52	22.54	0.64	9.59	2.28	2.75	-0.95	Implausible: <8% fat
9	87.4	197.2	22.47	89.66	21.21	0.60	8.67	-2.26	-2.59	0.66	Implausible: FM ≤ 0
10	85.9	183.2	25.59	71.75	21.38	4.22	8.00	14.15	16.47	-0.01	Coherence check
11	116.5	206.4	27.35	94.40	24.08	-	8.12	22.10	18.97	-	Coherence check
12	94.4	198.3	24.01	94.40	24.01	-	9.02	0.00	0.00	-	Implausible: FM ≤ 0

13	81.0	189.5	22.56	81.00	22.56	-	10.13	0.00	0.00	-	Implausible: FM ≤ 0
14	100.8	196.0	26.24	96.93	25.54	1.10	8.98	3.87	3.84	-0.40	Implausible: <8% fat
15	83.3	180.0	25.71	83.50	23.38	-	7.92	-0.20	-0.24	-	Implausible: FM ≤ 0
16	107.9	211.3	24.17	99.25	22.29	2.80	6.91	8.65	8.02	-0.92	Coherence check

Notes: BMI = Body Mass Index; FFM = Fat-Free Mass; FFMI = Fat-Free Mass Index; FMI = Fat Mass Index; PhA = Phase Angle; FM = Fat Mass; BF = Body Fat. Hyphens (-) indicate missing or non-calculable data in the source file. Flags indicate mathematically implausible derived body composition values requiring coherence checks.

Table S4. Correlations between aerobic fitness (relative and absolute), partial correlations controlling for body mass, and per-game performance metrics.

Outcome	Relative MAS		Absolute		Partial (ctrl. body mass)		*n*
	ρ	*p*-value	*ρ*	*p*-value	*ρ*	*p*-value	
PTS/game	-0.691	0.0128	-0.448	0.1446	-0.738	0.0095	12
REB/game	-0.863	0.0003	-0.175	0.5868	-0.842	0.0011	12
AST/game	-0.112	0.7283	-0.762	0.0040	-0.402	0.2208	12
MIN/game	-0.586	0.0453	-0.713	0.0092	-0.725	0.0116	12
FG%	-0.572	0.0520	0.259	0.4168	-0.489	0.1266	12
PTS+REB+AST	-0.695	0.0122	-0.476	0.1182	-0.736	0.0099	12

Notes: *ρ* = correlation coefficient (Spearman's rank correlation or partial correlation); Relative = maximal aerobic speed from the shuttle run ($km \cdot h^{-1}$; rank-equivalent to the relative VO_2max estimate); Absolute = estimated aerobic power in $L \cdot min^{-1}$ (relative estimate × body mass); Partial = partial correlation assessing the relationship while statistically controlling for the effect of body mass. Bold values indicate statistically significant correlations ($p < 0.05$).

Table S5. Complete Spearman correlation (ρ) matrix between anthropometric/body-composition variables and per-game performance variables.

Anthropometric/Body-Composition Variable	Performance Variable	ρ	95% BCa CI	95% Fisher z CI	*p*-value	*n*	*q*-value (BH)
Stature	PTS/game	-0.16	[-0.70, +0.52]	[-0.64, +0.41]	0.584	14	0.819
Stature	REB/game	0.29	[-0.31, +0.74]	[-0.30, +0.72]	0.322	14	0.732
Stature	AST/game	-0.7	[-1.00, +0.01]	[-0.91, -0.20]	0.006	14	0.078
Stature	MIN/game	-0.33	[-0.75, +0.24]	[-0.74, +0.26]	0.253	14	0.721
Stature	FG%	0.28	[-0.46, +0.76]	[-0.31, +0.71]	0.342	14	0.732
Stature	PTS+REB+AST	-0.19	[-0.72, +0.47]	[-0.66, +0.39]	0.523	14	0.819
Body mass	PTS/game	-0.11	[-0.67, +0.61]	[-0.61, +0.45]	0.714	14	0.819
Body mass	REB/game	0.25	[-0.44, +0.74]	[-0.33, +0.69]	0.391	14	0.733
Body mass	AST/game	-0.6	[-0.82, -0.21]	[-0.87, -0.05]	0.022	14	0.169
Body mass	MIN/game	-0.28	[-0.74, +0.33]	[-0.71, +0.31]	0.333	14	0.732
Body mass	FG%	0.5	[-0.04, +0.84]	[-0.08, +0.83]	0.069	14	0.296
Body mass	PTS+REB+AST	-0.13	[-0.67, +0.57]	[-0.62, +0.43]	0.664	14	0.819
Arm span	PTS/game	0.26	[-0.34, +0.75]	[-0.33, +0.70]	0.375	14	0.733
Arm span	REB/game	0.49	[-0.11, +0.84]	[-0.09, +0.82]	0.076	14	0.306
Arm span	AST/game	-0.45	[-0.87, +0.23]	[-0.80, +0.13]	0.106	14	0.397
Arm span	MIN/game	0.08	[-0.45, +0.55]	[-0.47, +0.59]	0.788	14	0.819
Arm span	FG%	0.28	[-0.44, +0.82]	[-0.31, +0.71]	0.342	14	0.732
Arm span	PTS+REB+AST	0.21	[-0.43, +0.68]	[-0.37, +0.67]	0.474	14	0.806
BMI	PTS/game	0.08	[-0.37, +0.68]	[-0.47, +0.59]	0.782	14	0.819
BMI	REB/game	0.26	[-0.38, +0.76]	[-0.33, +0.70]	0.379	14	0.733

BMI	AST/game	-0.2	[-0.62, +0.37]	[-0.67, +0.37]	0.483	14	0.806
BMI	MIN/game	-0.09	[-0.52, +0.46]	[-0.59, +0.47]	0.771	14	0.819
BMI	FG%	0.69	[+0.19, +0.89]	[+0.18, +0.91]	0.007	14	0.078
BMI	PTS+REB+AST	0.1	[-0.38, +0.72]	[-0.46, +0.60]	0.737	14	0.819
Σ8 skinfolds	PTS/game	-0.14	[-0.69, +0.61]	[-0.63, +0.43]	0.637	14	0.819
Σ8 skinfolds	REB/game	-0.05	[-0.62, +0.58]	[-0.57, +0.50]	0.87	14	0.87
Σ8 skinfolds	AST/game	-0.7	[-0.88, -0.32]	[-0.91, -0.20]	0.006	14	0.078
Σ8 skinfolds	MIN/game	-0.26	[-0.74, +0.34]	[-0.70, +0.33]	0.374	14	0.733
Σ8 skinfolds	FG%	0.2	[-0.41, +0.64]	[-0.37, +0.67]	0.483	14	0.806
Σ8 skinfolds	PTS+REB+AST	-0.35	[-0.79, +0.34]	[-0.75, +0.24]	0.227	14	0.708
Endomorphy	PTS/game	0.07	[-0.48, +0.72]	[-0.48, +0.58]	0.805	14	0.819
Endomorphy	REB/game	0.11	[-0.47, +0.70]	[-0.45, +0.61]	0.714	14	0.819
Endomorphy	AST/game	-0.69	[-0.88, -0.32]	[-0.91, -0.18]	0.007	14	0.078
Endomorphy	MIN/game	-0.21	[-0.69, +0.35]	[-0.67, +0.37]	0.469	14	0.806
Endomorphy	FG%	0.44	[-0.25, +0.71]	[-0.15, +0.80]	0.118	14	0.416
Endomorphy	PTS+REB+AST	-0.12	[-0.64, +0.51]	[-0.61, +0.44]	0.692	14	0.819
Mesomorphy	PTS/game	0.31	[-0.22, +0.71]	[-0.28, +0.73]	0.281	14	0.732
Mesomorphy	REB/game	0.34	[-0.21, +0.78]	[-0.25, +0.74]	0.236	14	0.708
Mesomorphy	AST/game	0.15	[-0.43, +0.69]	[-0.42, +0.63]	0.615	14	0.819
Mesomorphy	MIN/game	0.17	[-0.39, +0.58]	[-0.40, +0.64]	0.568	14	0.819
Mesomorphy	FG%	0.56	[-0.03, +0.87]	[-0.01, +0.85]	0.039	14	0.212
Mesomorphy	PTS+REB+AST	0.34	[-0.23, +0.80]	[-0.25, +0.75]	0.233	14	0.708
Ectomorphy	PTS/game	-0.29	[-0.68, +0.27]	[-0.72, +0.30]	0.318	14	0.732

Ectomorphy	REB/game	-0.18	[-0.71, +0.43]	[-0.65, +0.39]	0.537	14	0.819
Ectomorphy	AST/game	-0.13	[-0.68, +0.48]	[-0.62, +0.44]	0.67	14	0.819
Ectomorphy	MIN/game	-0.11	[-0.52, +0.45]	[-0.60, +0.45]	0.719	14	0.819
Ectomorphy	FG%	-0.57	[-0.85, -0.05]	[-0.86, -0.01]	0.034	14	0.202
Ectomorphy	PTS+REB+AST	-0.28	[-0.73, +0.37]	[-0.71, +0.30]	0.326	14	0.732
Phase angle	PTS/game	-0.12	[-0.70, +0.48]	[-0.61, +0.44]	0.681	14	0.819
Phase angle	REB/game	-0.1	[-0.56, +0.50]	[-0.60, +0.46]	0.731	14	0.819
Phase angle	AST/game	0.13	[-0.57, +0.69]	[-0.43, +0.62]	0.659	14	0.819
Phase angle	MIN/game	-0.08	[-0.55, +0.48]	[-0.58, +0.48]	0.799	14	0.819
Phase angle	FG%	-0.57	[-0.83, -0.13]	[-0.86, -0.02]	0.032	14	0.202
Phase angle	PTS+REB+AST	-0.08	[-0.56, +0.53]	[-0.58, +0.47]	0.794	14	0.819
Maximal aerobic speed	PTS/game	-0.69	[-0.93, -0.19]	[-0.92, -0.12]	0.013	12	0.11
Maximal aerobic speed	REB/game	-0.86	[-0.98, -0.57]	[-0.97, -0.49]	<0.001	12	0.018
Maximal aerobic speed	AST/game	-0.11	[-0.69, +0.55]	[-0.65, +0.50]	0.728	12	0.819
Maximal aerobic speed	MIN/game	-0.59	[-0.88, -0.10]	[-0.88, +0.04]	0.045	12	0.226
Maximal aerobic speed	FG%	-0.57	[-0.87, -0.01]	[-0.88, +0.05]	0.052	12	0.24
Maximal aerobic speed	PTS+REB+AST	-0.69	[-0.94, -0.22]	[-0.92, -0.13]	0.012	12	0.11

Notes: ρ = Spearman's rank correlation coefficient; BCa = bias-corrected and accelerated bootstrap 95% confidence interval (2000 resamples); Fisher z = Fisher z-transformation 95% confidence interval; p = exact two-tailed p-value; q_BH = false discovery rate adjusted q-value using the Benjamini–Hochberg procedure across all 60 tests; n = sample size (n = 14 for anthropometric variables, n = 12 for Maximal aerobic speed due to shuttle-run completion). Statistically significant p-values ($p < 0.05$) and q-values ($q < 0.05$) are formatted in bold.

Table S6. Descriptive anthropometric, body-composition and per-game characteristics by positional group.

Group	Bigs		Guards		Wings/Forwards	
**Variable**	**Mean ± SD**	***n***	**Mean ± SD**	***n***	**Mean ± SD**	***n***
Stature	203.00 ± 5.68	6	188.67 ± 6.29	9	198.30 ± -	1
Body mass	107.60 ± 9.11	6	82.70 ± 4.15	9	94.40 ± -	1
BMI	26.10 ± 1.74	6	23.30 ± 1.93	9	24.01 ± -	1
Σ8 skinfolds	92.05 ± 18.98	6	78.00 ± 34.33	9	64.80 ± -	1
Phase angle	8.36 ± 0.85	6	8.99 ± 0.83	9	9.02 ± -	1
Maximal aerobic speed	14.64 ± 1.14	5	15.71 ± 1.15	7	15.80 ± -	1
MIN/game	16.30 ± 5.46	5	16.57 ± 5.41	8	7.50 ± -	1
PTS/game	10.44 ± 6.10	5	8.33 ± 5.72	8	3.00 ± -	1
REB/game	5.00 ± 2.11	5	2.42 ± 1.03	8	1.00 ± -	1
AST/game	1.30 ± 0.85	5	2.55 ± 1.20	8	1.50 ± -	1
FG%	67.12 ± 10.29	5	52.08 ± 10.55	8	33.30 ± -	1

Notes: SD = Standard Deviation. For the Wings/Forwards group, SD is not applicable (-) as the sample size is n = 1. Anthropometric and body-composition variables correspond to baseline assessments; per-game characteristics are derived from the competition box-score data.
